# BA-ARAP-NMA: A Local Geometry Control Nonlinear Normal-Mode Analysis Method

**DOI:** 10.3390/biology15171533

**Published:** 2026-09-03

**Authors:** Zhenyu Zhang, Dejian Liu, Haiying Yu, Luyan Z. Ma

**Affiliations:** 1 State Key Laboratory of Microbial Diversity and Innovative Utilization, Institute of Microbiology, Chinese Academy of Sciences, Beijing 100101, China; zhangzhenyu222@mails.ucas.ac.cn (Z.Z.); yuhy@im.ac.cn (H.Y.); 2 University of Chinese Academy of Sciences, Beijing 100049, China

**Keywords:** normal-mode analysis, elastic network model, Cα model, protein conformational change, ARAP, nonlinear structure generation

## Abstract

Low-frequency normal-mode analysis (NMA) is widely used to predict collective protein motions, but direct Cartesian scaling progressively distorts the alpha-carbon (Cα) backbone as the amplitude increases. We present backbone-angle-compatible as-rigid-as-possible normal-mode analysis (BA-ARAP-NMA), a nonlinear Cα structure-generation method that constrains first-order changes in adjacent and next-nearest Cα distances during anisotropic network model (ANM) calculations and applies a co-rotational as-rigid-as-possible (ARAP) correction during structure generation. Tests on 35 experimentally characterized two-state protein pairs showed that the geometric constraints retained most of the input-to-second-state displacement information. In 34 of 35 pairs, recalculating the constrained modes increased the concentration of the retained transition information in the leading low-frequency modes. Across matched displacements, BA-ARAP-NMA substantially reduced local Cα distance and angle distortions in every pair. At the protein-pair level, BA-ARAP-NMA generally yielded lower root mean square deviation (RMSD) to the paired state and higher transition coverage than linear Cα-ANM while maintaining improved virtual-angle accuracy. BA-ARAP-NMA therefore extends Cα normal-mode structure generation beyond direct linear scaling, retaining transition-related collective information while providing amplitude-ordered structures with frame-level geometric measurements for subsequent rebuilding and refinement.

## Simple Summary

Proteins are important molecules carrying out essential biological functions, and their functions are highly related to conformational changes. Because it is experimentally difficult to capture these dynamic processes, scientists often rely on computational methods to explore these transformations. However, simulating large structural changes often causes the computed protein structure to become locally distorted. To address this, we improved a widely used computational method by adding geometric controls both when movement directions are calculated and when new structures are generated. Tests on 35 pairs of experimentally determined protein structures showed that our refined calculation substantially reduced local distortion while retaining the large-scale movement information provided by conventional predictions. Rather than returning just a single result, our tool produces a series of structural models with different degrees of change. By generating these structures with better local geometry, this method helps scientists better investigate protein functional mechanisms, ultimately contributing to a deeper fundamental understanding of biological processes.

## 1. Introduction

Proteins, as molecular units carrying out essential biological functions, rely on their specific three-dimensional architectures [1]. Through evolution, protein structures also encode dynamic information, including hinge sites and flexible regions that support transitions between functional states. Consequently, an experimentally determined structure provides a computational basis for exploring possible global conformational changes.

To computationally decode this dynamic information, normal-mode analysis (NMA) combined with elastic network models (ENMs) has long served as a foundational approach [2,3,4]. Rather than modeling every individual atom in full detail, modern ENMs typically coarse-grain the protein as a network of alpha-carbon (Cα) atoms connected by harmonic springs [2,3,4]. When NMA is applied to this simplified Cα network, it calculates the intrinsic patterns of motion permitted by the structure. The lowest-frequency modes correspond to collective movements that the elastic network can undergo most readily, often involving large groups of residues or entire domains. Because these motions can pivot around flexible regions and hinge sites encoded within the fold, they often capture principal directions of large-scale structural transitions [5,6,7,8].

Despite its predictive power for the direction of collective motion, standard NMA has a practical limitation when it is used to generate structural models of large conformational changes. Mathematically, standard NMA is based on linear approximations of infinitesimal displacements around a local energy minimum [2,3,4]. To model a macroscopic conformational change, researchers typically extrapolate these displacements by multiplying the movement specified by a selected low-frequency mode and adding it to the input Cα coordinates. However, when the displacement along the selected mode is enlarged by several angstroms (Å), this linear extrapolation can progressively disrupt the local geometry of the Cα network. As the requested amplitude increases, adjacent Cα distances, which form the virtual Cα bonds, can be artificially stretched or compressed, and the corresponding virtual angles can become increasingly distorted [9,10,11,12]. The overall predicted rearrangement may remain recognizable even as the underlying Cα trace undergoes substantial geometric degradation. These distortions complicate subsequent all-atom rebuilding and limit the direct use of generated intermediate structures as starting models for further simulations [13].

To reduce this geometric degradation, previous studies have developed strategies that generally follow two routes. The first route avoids direct Cartesian extrapolation by changing how motion is represented. Nonlinear rigid-block normal-mode analysis (NOLB) propagates low-frequency movements through finite rotations and translations of rigid blocks [9]. Internal-coordinate formulations, including iMODS, describe motion through torsion angles, allowing bond lengths and bond angles to be retained without directly scaling Cartesian coordinates [10,11,12]. The second route introduces geometric restrictions during structure generation. CONCOORD derives distance constraints from an experimental structure and generates conformations that satisfy those constraints [14]. FRODA combines rigidity analysis with constrained geometric simulation [15], whereas NMSim uses normal-mode directions to guide constrained simulations of conformational change [16].

Despite their different implementations, these approaches share the goal of preserving local geometry while sampling large-scale conformational change [9,12,14,15,16]. Inspired by this general principle, we explored a distinct strategy that retains a Cartesian Cα representation, avoids predefined rigid blocks, and introduces local geometric control during both mode calculation and structure generation.

In a conventional Cα elastic network, all contacts within a spatial cutoff are represented by the same harmonic spring form [2,3,4]. Adjacent and next-nearest Cα contacts are therefore not treated as a distinct class, even though they directly determine the geometry of the local Cα backbone. Backbone-angle-compatible as-rigid-as-possible normal-mode analysis (BA-ARAP-NMA) addresses this limitation by constraining first-order changes in adjacent and next-nearest Cα distances during mode calculation. The low-frequency modes are then recalculated in the resulting admissible space. Local stretching is suppressed without freezing the overall motion of the protein: the structure is not divided into rigid blocks, and coordinated rotations and changes in relative domain orientation can still develop within the recalculated modes.

During finite-amplitude structure generation, a co-rotational as-rigid-as-possible (ARAP) correction provides a second level of geometric control [17,18]. The local distance constraints shape the initial low-frequency modal basis, whereas the ARAP correction reduces the residual local drift that develops when a selected mode is extended to larger amplitudes.

To evaluate BA-ARAP-NMA, we used 35 experimentally determined two-state protein pairs spanning diverse collective motions. Because the local constraints alter the modal calculation, improved local geometry alone would be insufficient if the constraints removed transition-related collective motion. We therefore tested whether first-order local geometric constraints reduce Cα distortion while preserving experimentally observed two-state information. The evaluation first examined modal-space behavior, four-way ablation, and matched-displacement structure generation. It then assessed geometry-controlled reach and candidate-set size. Starting-structure sensitivity, cutoff robustness, and performance relative to NOLB were also evaluated. The paired experimental structure served only as an evaluation reference. It was excluded from mode calculation, structure generation, and displacement matching.

For practical use, these corrected motions must be returned as structures that can be examined rather than only as a theoretical endpoint. BA-ARAP-NMA therefore generates an amplitude-ordered series of Cα structures for each explored mode rank and orientation, together with frame-level measurements of local geometry. These outputs show how collective motions develop with amplitude and allow alternative mode branches to be compared. They also support the selection of intermediate models for all-atom rebuilding, refinement, or integration with independent experimental information [13].

## 2. Materials and Methods

### 2.1. Cα Representation and Input Structures

Our computational representation is the Cα trace, and each amino-acid residue is represented by its Cα atom. All local constraints were constructed only within a continuous segment and were not built across segment breaks caused by a change in chain identifier or a discontinuity in residue number.

Notation. Coordinate vectors and matrices are distinguished by context; $R_{\mathrm{rb}}$ denotes the orthonormal rigid-body basis, whereas $A_{\mathrm{ARAP}}$ denotes the nonlinear ARAP coordinate-mapping operator. Euclidean-orthonormal modal vectors $\phi_{k}$ are used in overlap calculations, and their unit-Cα-RMS forms $\hat{\phi_{k}}$ are used for structure generation.

For each two-state protein pair, common modeled Cα residues were mapped between the input and paired second-state structures. When a Protein Data Bank (PDB) entry contained multiple crystallographic copies of the same protein chain, the copy providing the most complete one-to-one Cα mapping was selected [19].

Specifically, we selected 1NQJ chain B in the collagenase pair because it retains the N-terminal linker residues included in the common-Cα mapping, whereas chain A is missing a larger portion of this segment [20].

The Cα coordinates of the input structure are written as:

$$X^{0}={\left[x_{1}^{0},x_{2}^{0},\ldots ,x_{N}^{0}\right]}^{T}\in R^{3N}$$

where N is the number of Cα nodes used in the calculation.

### 2.2. Cα Elastic Network Model

The input Cα trace is converted into a single-parameter anisotropic network model. Two Cα nodes are connected by a spring when their distance in the input structure is not larger than the cutoff radius. Primary benchmark calculations used $r_{c}$ = 15 Å [2,3,4]; the robustness analysis repeated the complete network construction at $r_{c}$ = 12 and 18 Å.

$$\Gamma_{ij}=1\;\mathrm{if}i\neq j\mathrm{and}\left\|x_{i}^{0}-x_{j}^{0}\right\|\leq r_{c};\;\;\Gamma_{ij}=0\;\mathrm{otherwise}$$

The ANM potential is as follows:

$$V_{\mathrm{ANM}}\left(X\right)=\frac{\gamma }{2}\sum_{i<j}\Gamma_{ij}{\left(\left\|x_{i}-x_{j}\right\|-d_{ij}^{0}\right)}^{2}$$

$$d_{ij}^{0}=\left\|x_{i}^{0}-x_{j}^{0}\right\|$$

Here, γ is a uniform spring constant. Our method uses eigenvector ordering and normalized modal directions, so the absolute value of γ does not change the geometry of the generated structures. At the linearized input structure, the off-diagonal Hessian block for connected nodes i and j is as follows:

$$H_{ij}=-\gamma e_{ij}e_{ij}^{T}$$

$$e_{ij}=\frac{x_{i}^{0}-x_{j}^{0}}{d_{ij}^{0}}$$

The diagonal block is assembled by the zero-row-sum condition:

$$H_{ii}=-\sum_{j\neq i}H_{ij}$$

### 2.3. Removal of Rigid-Body Motions

In normal-mode analysis, zero eigenvalues of the stiffness matrix arise from motions that leave the structure undeformed. The corresponding null modes therefore do not describe internal conformational changes [21]. When a protein Cα trace is considered as a whole in three-dimensional space, it has six rigid-body degrees of freedom: three translations along the Cartesian axes and three rotations about them. These motions translate or rotate the entire Cα trace and were removed.

Specifically, for the Cα model used here, this six-dimensional set of rigid-body motions was constructed analytically. The three translational fields were uniform displacements of all Cα nodes. The three rotational fields were defined with respect to the Cα centroid $\hat{x}$. For a rotation axis ω, the displacement of node i was as follows:

$$r_{i}\left(\omega \right)=\;\omega \times \left(x_{i}^{0}-\bar{x}\right)$$

After orthonormalization, these six displacement fields were projected out of the admissible modal space. The retained modes therefore describe internal Cα deformation rather than overall translation or rotation of the input structure.

### 2.4. Local Cα Constraints in Mode Calculation

When Cartesian normal modes are scaled to larger amplitudes, local geometry can be distorted because each mode is defined as an infinitesimal displacement at the input structure. Similar concerns have motivated nonlinear normal-mode methods and internal-coordinate formulations, in which bond-length and bond-angle distortions are reduced by changing how the motion is represented or generated [9,10,12,16]. In the present Cα model, the local quantities that can be constrained directly are distances between neighboring Cα nodes. We therefore imposed first-order constraints on adjacent and next-nearest Cα distances during mode calculation.

For each continuous Cα segment, the local pair set was defined as follows:

$$E_{\mathrm{loc}}=\{\left(i,i+1\right)\}\;\cup \;\{\left(i,i+2\right)\}$$

For a continuous segment containing n Cα nodes, the adjacent set uses i=1,…,n−1 and the next-nearest set uses i=1,…,n−2. The i,i+1 distance defines the length of a virtual Cα bond. The i,i+2 distance, together with the two adjacent virtual-bond lengths, determines the virtual angle formed by three consecutive Cα nodes. Preserving these distances to first order therefore removes first-order changes in local virtual-bond lengths and virtual angles from the modal displacement.

For a local pair (i,j), the distance-deviation function was defined as follows:

$$g_{ij}\left(X\right)=\left\|x_{i}-x_{j}\right\|-d_{ij}^{0}$$

Let u denote an infinitesimal displacement of the complete Cα coordinate vector, so that we find the following:

$$X=X^{0}+u$$

The first-order change in the local distance is obtained from the corresponding Jacobian row. Preserving this distance to first order therefore requires the following:

$$J_{ij}u=0$$

The nonzero Jacobian blocks are as follows:

$$\frac{\partial g_{ij}}{\partial x_{i}}=e_{ij}^{T}\;,\;\frac{\partial g_{ij}}{\partial x_{j}}=-e_{ij}^{T}$$

All local constraint rows were assembled into J. Using the rigid-body basis $R_{\mathrm{rb}}$ defined above, the admissible modal space was specified by the combined constraint matrix, as follows:

$$A=\left[\begin{array}{c} J\\ {R_{\mathrm{rb}}}^{T} \end{array}\right]$$

An orthonormal basis P of the null space of A was computed, as follows:

$$AP=0\;,\;P^{T}P=I$$

The ANM Hessian was then projected into this constrained space, as follows:

$$H_{c}=P^{T}HP$$

and the reduced eigenvalue problem was solved as follows:

$$H_{c}\psi_{k}=\lambda_{k}\psi_{k}$$

The Cartesian Cα mode was recovered by the following:

$$\phi_{k}=P\psi_{k}$$

Thus, each retained mode satisfies the following:

$$J\phi_{k}=0\;,\;{R_{\mathrm{rb}}}^{T}\phi_{k}=0$$

Taken together, the local-distance and rigid-body conditions determine which internal Cα motions enter the eigenvalue calculation. The Hessian is projected before diagonalization, so BA-ARAP-NMA recalculates the mode directions under these conditions instead of deleting selected modes from a completed linear Cα-ANM calculation; because the matrix being diagonalized has changed, their ordering can change as well. The resulting modes are the lowest-frequency internal motions that keep the selected Cα distances unchanged to first order, while coordinated rotations and changes in relative domain orientation remain possible. For N mapped Cα nodes distributed across S continuous segments, the local set contains 2N−3S constraint rows. These rows were full rank in all 70 input-state calculations; the local constraint set is defined entirely by the mapped Cα trace and therefore does not depend on the ANM cutoff. After the six global rigid-body modes were removed, the constrained nullity was N+3S−6; across the benchmark the median was 235 (range, 111–991), corresponding to 33.2% (range, 33.1–33.9%) of the unconstrained 3N−6 internal Cartesian dimension. The eigenvalues of $P^{T}HP$ order the motions within this restricted subspace and were used only to rank modes and were not interpreted as calibrated physical frequencies or used for thermal-amplitude or B-factor weighting.

### 2.5. Sampling Cα Structures Along Low-Frequency Modes

After the constrained modes were calculated, they were ordered by increasing eigenvalue. The position of a mode in this ordering is referred to as its mode rank. Rank 1 is the lowest-frequency retained internal mode, rank 2 is the next, and so forth. In the benchmark, the first ten mode ranks were used.

An eigenvector and its negative describe the same modal axis, so both orientations were sampled, as follows:

s∈{−1,+1}.

Here, s=+1 follows the eigenvector as stored, whereas s=−1 follows the opposite orientation.

For modal overlap calculations, $\phi_{k}$ denotes the Euclidean-orthonormal eigenvector returned by the projected eigenproblem. For coordinate generation, the same direction was rescaled to the unit-Cα-RMS vector $\hat{\phi_{k}}$, as follows:

$$\hat{\phi_{k}}\;=\;\frac{\phi_{k}}{{\left[N^{-1}\;\sum_{i}{\left\|\phi_{k,i}\right\|}^{2}\right]}^{\frac{1}{2}}}.$$

The requested amplitude $a_{t}$ sets the displacement scale along $\hat{\phi_{k}}$. Before ARAP correction, the linear predictor is as follows:

$$Y\left(a_{t}\right)=X^{0}+s\;a_{t}{\hat{\phi }}_{k}.$$

At this stage, $Y\left(a_{t}\right)$ is the coordinate set obtained by direct mode scaling. The ARAP map $A_{\mathrm{ARAP}}$ then produces the final Cα coordinates, as follows:

$$X\left(a_{t}\right)=A_{\mathrm{ARAP}}\left(X^{0},Y\left(a_{t}\right)\right).$$

For a fixed mode rank k and orientation s, the same initial mode vector was used at every requested amplitude. Increasing $a_{t}$ changed the displacement magnitude without recalculating the mode.

### 2.6. Co-Rotational ARAP Mapping

Once the linear predictor provides the Cα coordinates derived from scaling a selected normal mode, BA-ARAP-NMA applies a geometric correction based on the as-rigid-as-possible (ARAP) idea [17,18].

The ARAP correction was defined on the local Cα edge set $E_{\mathrm{loc}}$. For each Cα node i, let $N_{i}$ denote the set of its local neighbors in this edge set. The local rotation $Q_{i}$ was fitted by comparing the local edge vectors in the input trace with the corresponding edge vectors in the linear predictor, as follows:

$$Q_{i}=\underset{Q\in \mathrm{SO}\left(3\right)}{\arg\;\min}\sum_{j\in N_{i}}{∥\left(y_{j}-y_{i}\right)-Q\left(x_{j}^{0}-x_{i}^{0}\right)∥}^{2}.$$

This orthogonal Procrustes fit was solved by singular-value decomposition with determinant correction [22,23]. The principal matrix logarithm of the fitted rotation, defined below, corresponding to a fitted rotation angle in [0, π), gives the first-order rotational part. The implementation stops if a fitted rotation approaches π, where the principal logarithm becomes ambiguous. No generated benchmark frame triggered this guard.

$$\Omega_{i}=\log\left(Q_{i}\right),$$

and the remaining finite-rotation term was defined as follows:

$$B_{i}=Q_{i}-I-\Omega_{i}.$$

This construction retains the first-order modal displacement and adds the higher-order rotational correction produced by mode scaling. Locally, $y_{j}-y_{i}\approx \left(I+\Omega_{i}\right)\left(x_{j}^{0}-x_{i}^{0}\right)$; adding $B_{i}\left(x_{j}^{0}-x_{i}^{0}\right)$ changes the target edge to $Q_{i}\left(x_{j}^{0}-x_{i}^{0}\right)$. Thus, a pure local rotation preserves the reference-edge length exactly.

For a local edge $\left(i,j\right)$, the corrected target edge vector was as follows:

$$t_{ij}^{\mathrm{ARAP}}=\left(y_{i}-y_{j}\right)+\frac{1}{2}\left(B_{i}+B_{j}\right)\left(x_{i}^{0}-x_{j}^{0}\right).$$

The final Cα coordinates were then obtained by finding the global trace whose local edge vectors best matched these ARAP-corrected targets, as follows:

$$X\left(a_{t}\right)\;=\;{\arg\;\min}_{Z}\;\sum_{\left(i,j\right)\;\in \;E_{\mathrm{loc}}}{\left\|z_{i}-z_{j}-t_{ij}^{\mathrm{ARAP}}\right\|}^{2}.$$

Because the local edge set was constructed separately within each continuous Cα segment, an input containing more than one segment produced a disconnected local graph. The graph Laplacian consequently had an independent translational null space for each connected component. In the Karush–Kuhn–Tucker (KKT) system, the three Cartesian coordinates of each component centroid were constrained to those of the corresponding component in the linear predictor, thereby removing arbitrary component-wise translation without introducing interactions between separate segments. The implementation applied this map once per generated frame. Local rotations were fitted from the linear predictor, followed by a single component-gauged KKT solve. No iterative alternation between local and global steps was used. The sparse factorization depends only on the local graph and segment pattern and was reused across amplitudes for the same input.

### 2.7. Linear Cα-ANM Baseline

Linear Cα-ANM was used as the baseline for comparison. Its modes were calculated from the standard Cα-ANM Hessian after the rigid-body motions were removed, without the local-distance projection used in BA-ARAP-NMA. After normalization, the coordinates were obtained by direct Cartesian scaling, as follows:

$$X_{\mathrm{lin}}\left(a_{t}\right)=X^{0}+s\;a_{t}\;{\hat{\phi }}_{k}^{\mathrm{ANM}}.$$

The two methods used the same input structure, mode ranks, orientations, and amplitude values. The local geometry metrics and retrospective second-state metrics were calculated in the same way.

### 2.8. Test Systems and Validation Design

The evaluation included 35 experimentally determined protein structure pairs for which a one-to-one common-Cα representation could be established. Each experimental structure in a pair was used separately as the input for a complete calculation, so the Hessian, modal basis, and generated structures were recalculated from both starting states. The two input-state calculations remained nested within the same protein pair and were not treated as independent proteins. All 35 pairs underwent the same modal-space, four-way ablation, and matched-displacement analyses. The same systems were also used for local geometry, retrospective, and cutoff evaluations. The complete benchmark is reported in Table A1.

The benchmark was designed to span protein sizes and structural-motion classes while limiting family redundancy. Eligible pairs required a one-to-one common-Cα mapping and at least 95% sequence identity. Each structure also had to provide at least 85% modeled-Cα coverage. The mapped representation contained 80–1200 common Cα residues, had a common-Cα root mean square deviation (RMSD) of at least 3 Å, and included a dominant continuous segment covering at least 70% of each state. Finally, the 15 Å ANM network had to be connected, and mode ranks 1–10 had to be calculated successfully from both experimental inputs. Pairs were drawn from a published nonredundant collection of experimentally determined structure pairs [24]. Additional pairs came from primary studies of chaperone cycles and allosteric switches, as well as membrane transport, fold switching, and mechanosensitive gating. Comparative performance values from BA-ARAP-NMA, linear Cα-ANM, and NOLB were not used to select pairs.

The resulting benchmark contained 111–994 common Cα nodes per pair, with a median of 238. Common-Cα RMSD ranged from 3.627 to 34.384 Å (median, 9.533 Å), sequence identity ranged from 97.2% to 100%, and the minimum mapped coverage was 94.9%. The represented motions included domain closure or reorientation, enzyme and chaperone cycles, and nucleic-acid-associated rearrangements. The benchmark also covered alternating-access transport, fold switching, and mechanosensitive-channel gating. Detailed eligibility, exclusion, and residue-mapping information is provided in Supplementary File S1.

The paired experimental structure was excluded from mode calculation and deformation design. It was also excluded from ARAP mapping, structure generation, and matched-displacement selection. It was introduced only for evaluation: cumulative overlap was calculated from the experimental displacement, and generated structures were scored by paired-state RMSD and transition coverage. The same fixed structure sets were used for the retrospective minimum-RMSD analysis. Thus, the matched-displacement and local geometry analyses were selected independently of the paired structure, whereas the minimum-RMSD analyses quantified the best candidate contained in fixed, equal-sized structure sets.

### 2.9. Sampling Grid and Four-Way Ablation Design

For every protein pair, each experimental structure was used in turn as the input. For each input state, the first ten non-rigid mode ranks were sampled in both orientations. Four matched variants were compared: linear Cα-ANM with direct Cartesian scaling; linear Cα-ANM followed by ARAP mapping; BA-constrained modes with direct scaling; and the complete BA-ARAP-NMA method. All variants used the same input coordinates, mode ranks, and orientations. They also shared the deformation amplitude values and evaluation procedures. Across the complete 35-pair benchmark, this yielded 35 pairs × 2 input structures × 10 mode ranks × 2 orientations = 1400 branches per method.

The deformation amplitude grid was $a_{t}$ = 0, 0.25, 0.50, …, 20.00 Å, including the input structure at 0 Å. A frame denotes the structure generated at one amplitude along a fixed mode rank and orientation; it is an amplitude index, not physical time. Coordinates were generated for every frame and used immediately to calculate the structural metrics. Frame-level numerical results were retained for protein-pair-, input-state-, and branch-level analysis, whereas bulk coordinate trajectories were not written for the large-scale benchmark.

### 2.10. Cumulative Overlap Analysis

For each protein pair, the experimentally observed Cα displacement was calculated from the common Cα coordinates of the input structure and the rigidly aligned paired second-state structure, as follows:

$$\Delta R=\mathrm{vec}\left(X_{\mathrm{second}}^{\mathrm{aligned}}-X^{0}\right).$$

The displacement vector was normalized as follows:

$$\hat{\Delta R}=\frac{\Delta R}{\left\|\Delta R\right\|}.$$

The modes in each modal basis were Euclidean-orthonormal. The overlap of mode $\phi_{k}$ with the observed displacement was therefore as follows:

$$o_{k}=\phi_{k}^{T}\hat{\Delta R},$$

and the cumulative overlap of the first K modes was calculated as follows:

$$\mathrm{CO}\left(K\right)={\left(\sum_{k=1}^{K}{o_{k}}^{2}\right)}^{\frac{1}{2}}.$$

The calculation was performed separately for the standard Cα-ANM modes and the BA-ARAP-NMA constrained modes. Because the structure-generation benchmark used the first ten low-frequency mode ranks, K=10 was the primary comparison. Values from K=1 to K=50 were also evaluated to determine whether the result depended on the number of modes included in the low-frequency modal set [7,25].

To distinguish the amount of the observed displacement retained after applying the constraints from its concentration in the leading modes, we calculated two additional quantities. Let $\hat{\Delta R}$ denote the normalized input-to-second-state Cα displacement defined above. The retained fraction was calculated as follows:

$$\eta ={∥P^{T}\hat{\Delta R}∥}^{2}.$$

The quantity $\sqrt{\eta }$ gives the maximum cumulative overlap attainable when the complete set of constrained modes is included. For linear Cα-ANM, the corresponding value was calculated from its complete rigid-body-free mode set.

To measure how strongly the retained displacement was concentrated in the first K modes, we calculated the following:

$$\rho_{K}=\frac{\mathrm{CO}{\left(K\right)}^{2}}{\eta },$$

where $\mathrm{CO}\left(K\right)$ is the cumulative overlap of the first K modes and η is the retained fraction calculated for the same method. Together, these quantities describe how much of the observed displacement is retained after the local distance constraints are applied and how strongly that retained motion is concentrated in the leading modes. They are not direct measures of low-frequency performance relative to linear Cα-ANM, which was evaluated separately by cumulative overlap at matched mode counts.

To quantify the local distance change contained in the experimentally observed transition, ΔR was scaled to a global Cα-RMS magnitude of 1 Å, as follows:

$$\Delta R^{\left(1\;Å\right)}=\frac{\Delta R}{{\mathrm{RMSD}}_{C\alpha }\left(\Delta R\right)},$$

and the root mean square first-order change in the constrained local distances was calculated as follows:

$$s_{\mathrm{loc}}=\sqrt{\frac{1}{m}{∥J\Delta R^{\left(1\;Å\right)}∥}^{2}},$$

where m is the number of local distance constraints.

### 2.11. Generated-Structure Evaluation Metrics

Generated Cα structures were evaluated by local geometry and retrospective comparison with the paired second-state structure.

For local geometry, we first compared the virtual Cα backbone of each generated structure $X_{t}$ with that of the input structure $X^{0}$. For each adjacent Cα pair, the distance deviation was calculated as follows:

$$\Delta d_{i,t}=d_{i,i+1}\left(X_{t}\right)-d_{i,i+1}\left(X^{0}\right)$$

The mean absolute value of $\Delta d_{i,t}$ was reported as adjacent Cα distance mean absolute error (MAE). The same set of deviations was also used to calculate the mean squared adjacent Cα distance deviation, as follows:

$$D_{\mathrm{adj}}^{2}\left(X_{t}\right)=\frac{1}{M_{\mathrm{adj}}}\sum_{\left(i,i+1\right)\in E_{\mathrm{adj}}}{\left[d_{i,i+1}\left(X_{t}\right)-d_{i,i+1}\left(X^{0}\right)\right]}^{2}.$$

where $E_{\mathrm{adj}}$ is the set of adjacent Cα pairs within continuous segments and $M_{\mathrm{adj}}=\left|E_{\mathrm{adj}}\right|$ is the number of such pairs. Because this metric is based on squared deviations, strongly stretched or compressed adjacent Cα pairs contribute more prominently than in the MAE. Virtual-angle error was calculated from consecutive Cα triplets. The angle in $X_{t}$ was compared with the corresponding angle in $X^{0}$, and the mean absolute angular deviation was reported as virtual-angle MAE. In addition, adjacent Cα distance and virtual-angle outlier counts were recorded by counting local pairs or triplets whose deviations exceeded the reporting thresholds. Adjacent Cα distance outliers were counted at absolute deviations greater than 0.5 Å and 1.0 Å, and virtual-angle outliers were counted at deviations greater than 10°. The mean-squared quantity $D_{\mathrm{adj}}^{2}$ is retained in the frame-level output table in Supplementary File S1 as a distortion-sensitive diagnostic; adjacent-distance MAE is the primary main-text distance endpoint.

For retrospective comparison with the paired second-state structure, each generated structure was rigidly aligned to that structure [22,23]. The Cα RMSD at frame t was calculated as follows:

$${\mathrm{RMSD}}_{t}={\left[N^{-1}\sum_{i}{∥x_{i,t}^{\mathrm{aligned}}-x_{i,\mathrm{alt}}∥}^{2}\right]}^{1/2}$$

Transition coverage was defined as follows:

$$C_{t}=\frac{{\mathrm{RMSD}}_{0}-{\mathrm{RMSD}}_{t}}{{\mathrm{RMSD}}_{0}}$$

where ${\mathrm{RMSD}}_{0}$ is the aligned Cα RMSD between the input structure and the paired second-state structure. A positive $C_{t}$ indicates that the generated structure has a lower aligned Cα RMSD to the paired second-state structure than the input structure. The alternative-state coordinate in the RMSD expression is the corresponding mapped Cα coordinate from the paired second-state structure.

### 2.12. Matched-Displacement Pairing and Method Contrasts

The four-way ablation separated the effect of recalculating the modal basis from that of finite-amplitude ARAP mapping. Because ARAP can alter the final displacement, the primary comparisons used matched actual Cα displacements of 1, 2, 3, 5, 8, and 10 Å rather than relying on requested amplitude alone. Each mode rank and orientation defined one branch within a protein pair. For every target displacement, the closest generated structure was selected without reference to the paired experimental structure, and the same branch was used for all method contrasts. A branch–displacement contrast was retained only when the selected structure from each variant lay within 0.25 Å of the target. Otherwise, no contrast was calculated for that branch at that displacement. Table A12 summarizes the effect of tightening the tolerance from 0.25 to 0.10 Å. Complete matching counts and branch-level diagnostics are provided in Supplementary File S1.

### 2.13. Evaluation Without Paired-Structure-Based Selection

Two additional analyses examined generated structures without using the paired experimental structure during selection. First, for each branch defined by a mode rank and orientation, we recorded the largest actual displacement reached with zero adjacent-Cα distance outliers greater than 1 Å and zero virtual-angle outliers greater than 10°. This geometry-controlled reach directly tests the local distance and angle quantities that BA-ARAP-NMA is designed to control while remaining independent of the paired experimental structure during selection.

Second, the influence of candidate-set size was evaluated separately from the method effect. Duplicated zero-amplitude structures were excluded, leaving 1600 paired nonzero structure indices per method within each input-state calculation (20 branches defined by mode rank and orientation × 80 nonzero amplitudes). Subsets containing 1, 5, 10, 20, 40, 80, 160, 320, 640, or all 1600 paired indices were fixed before paired-state scoring. The lowest RMSD within each subset was then calculated. The 20-structure subset was the primary small-budget comparison. This analysis reports how retrospective minimum RMSD changes with the number of generated structures evaluated.

### 2.14. Comparison with NOLB Without Using the Paired Structure During Generation

NOLB version 1.9 was used as the nonlinear external comparator [9,26]. It was selected because, like BA-ARAP-NMA, it generates Cartesian protein structures from low-frequency collective modes and can be run without a paired target. Candidate sets could therefore be matched by actual Cα displacement and structure number. The comparison used target-free random-ensemble generation: NOLB received only the input structure, ten low-frequency modes, and the requested sampling scale, while the paired experimental structure was withheld during generation. NOLB structures were mapped to the same common-Cα representation used for BA-ARAP-NMA and linear Cα-ANM. Only structures whose actual aligned Cα displacement lay within ±0.20 Å of 1, 2, 3, 5, 8, or 10 Å were admitted to the comparison. NOLB used a 5.0 Å cutoff with ten modes. Nonlinear sampling was enabled with --nlin, and the requested distance was set with --dist 1. Each displacement pool began with 80 pilot models. Production then proceeded in batches of 500 until either at least 50 candidates were accepted or 16 batches had been completed. A minimum of 20 accepted candidates was required for analysis. Exact commands and run logs are provided in Supplementary File S1.

Because full target-free NOLB sampling was substantially more computationally intensive than the 35-pair Cα calculations, a 12-pair comparator set was selected with a performance-independent, diversity-stratified design. The 35 pairs were classified by common-Cα count (<200, 200–399, or ≥400 residues) and initial pair RMSD (<7, 7–<12, or ≥12 Å). Each of the seven occupied size-by-RMSD groups contributed at least one pair. The remaining five positions were allocated in proportion to group size, and MscS was included a priori to represent mechanosensitive gating. Within each group, preference was given to motion classes and protein families not yet represented. Remaining ties were resolved by a fixed order independent of method outputs. The resulting set spans all seven occupied groups and 12 distinct motion classes (Table A9). No performance measure from BA-ARAP-NMA, linear Cα-ANM, or NOLB was used in selection. Both experimental structures were used separately as input. The primary comparison used 20 structures per method. For linear Cα-ANM and BA-ARAP-NMA, these were the ten mode ranks sampled in both orientations. For NOLB, 20 structures were sampled from each strict matched-displacement pool. Candidate sets of 1, 5, and 10 structures were retained as sensitivity analyses. The 12 pairs are indicated by a dagger in Table A1. Full allocation details are provided in Supplementary File S1.

In this study, target-free refers to structure generation and fixed candidate-set construction. After each set had been fixed, the paired experimental structure was used retrospectively to identify the minimum-RMSD candidate; the RMSD, transition coverage, and local geometry measurements of that candidate were then recorded. Equal structure numbers control the opportunity to obtain a favorable candidate from a fixed set and do not imply equal runtime or equal computational cost.

### 2.15. Statistical Analysis

The protein pair was the independent statistical unit. For each contrast, metric, and displacement, available branch effects were summarized by the median within each input-state calculation. The two input-state summaries were then combined by their median to yield one effect per protein pair. Branches were deterministic design conditions rather than independent biological replicates. Table A13 reports their distributions and a hierarchical sensitivity analysis that resampled branches within each input state before resampling protein pairs. Complete branch-level values and matching-quality records are provided in Supplementary File S1, whereas protein-pair-level intervals remain the primary inferential results. The two input states were also reported separately. Their absolute effect difference described starting-structure sensitivity without depending on reporting order. Primary results are presented as median paired effects with bias-corrected and accelerated (BCa) 95% confidence intervals from 10,000 protein-pair bootstrap resamples [27]. Exact two-sided sign tests evaluated the consistency of effect direction across protein pairs. Holm adjustment was applied across the six displacement levels within each metric-by-contrast family. Conceptually distinct endpoints were analyzed in separate families. Wilcoxon signed-rank tests, Hodges–Lehmann shifts, and rank-biserial effects were retained as sensitivity analyses in Supplementary File S1. For the K = 1–50 modal curve, a simultaneous bootstrap confidence band was used rather than 50 separate tests.

For the 12-pair NOLB comparison, candidate-set resampling was performed within each input-state calculation before the two input-state effects were combined at the protein-pair level. The analysis based on 20 structures per method was the primary comparison. The shaded range in the structure-number analysis is the 2.5–97.5% range across fixed paired structure subset resamples and is not a confidence interval for a protein population. BCa intervals quantify uncertainty in the median pair-level effect, whereas exact sign tests evaluate the consistency of effect direction across protein pairs.

### 2.16. Robustness and Computational Cost

Cutoff sensitivity was evaluated uniformly across all 35 pairs. At each cutoff of 12, 15, or 18 Å, linear Cα-ANM and complete BA-ARAP-NMA were repeated from both input structures. Each calculation used mode ranks 1–10, both orientations, and requested amplitudes of 0, 1, 3, 5, 10, and 20 Å. Unlike the primary matched-actual-displacement analysis, this robustness calculation used the requested-amplitude grid directly; its 15 Å values therefore need not equal the primary 15 Å matched-displacement estimates. Computational cost was measured in fresh processes as end-to-end wall time and peak resident memory. Because BA-ARAP-NMA returns Cα structures with frame-level quality measurements whereas NOLB includes calibration and full-atom random-ensemble sampling, the values describe the complete procedures rather than a pure algorithmic speed ranking. All timing runs used the same WSL2 Linux environment. The thread count was fixed at one for OpenBLAS, MKL, OpenMP, and NumExpr. The versions used for the reported study are stated in Section 2.17 and summarized in the Supplementary File S1 README under ‘Software versions used for the reported study’, with calculation-level records in the archived ACTIVE_ENVIRONMENT.json files.

### 2.17. Implementation and Output

BA-ARAP-NMA v1.0.0 was implemented for Python 3.10 or later using NumPy and SciPy for matrix operations, eigen decomposition, and sparse linear-system solution [28,29]. The constrained-mode calculation, ARAP mapping, and coordinate-generation procedures used the v1.0.0 implementation [30,31]. The mean-squared adjacent-Cα distance deviation defined in Section 2.11 was calculated with the accompanying analysis scripts. For each mode rank and orientation, the program writes a multi-model Cα PDB file and a connected Cα trace. It also produces frame-level and summary tables, with an optional PyMOL visualization script [32]. When the input contains non-Cα protein atoms, the software can write residue-local decorated full-atom PDB files. These files are intended for visualization and as starting points for rebuilding and refinement; they are not energy-minimized all-atom models. Supplementary File S1 provides the source code, analysis scripts, exact inputs, source data, software hashes, and reproducibility records. The reported calculations used Python 3.12.3, NumPy 2.3.5, SciPy 1.17.0, pandas 2.2.3, and Matplotlib 3.10.8; PyMOL v2.5 was used for optional molecular visualization.

Generative AI tools were used during manuscript preparation for language editing, assistance with debugging analysis and supporting scripts, organization of supporting files, and as a secondary aid in checking consistency among scripts, tabulated outputs, and manuscript text. The benchmark definition, input structures, analysis design, statistical procedures, thresholds, and scientific interpretation were determined by the authors. All calculations were performed using the scripts and source data provided in Supplementary File S1, and the authors independently checked and approved the numerical outputs, figures, tables, software, and Supplementary Materials.

## 3. Results

### 3.1. Local Cα Constraints Preserve Transition-Related Information Across the 35-Pair Benchmark

The first question was whether the local Cα constraints removed the collective motion that makes low-frequency NMA useful. For each pair, we compared the cumulative overlap between the experimentally observed displacement connecting the two mapped structures and the first K modes from linear Cα-ANM or the BA-constrained basis. Cumulative overlap therefore measures how well a low-frequency modal subspace represents the experimental structural difference rather than whether one mode reproduces the complete change.

**Figure 1:**
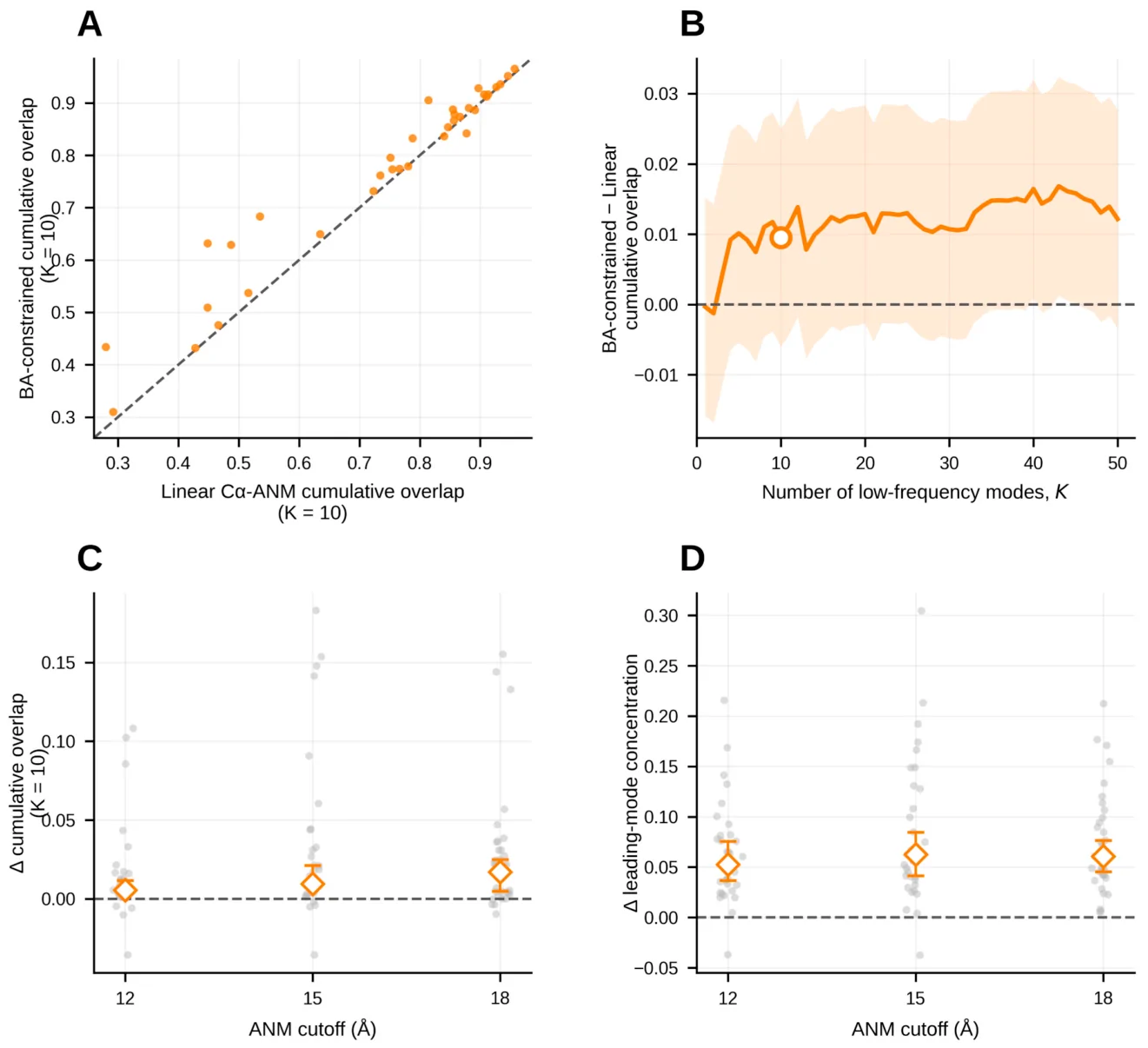
Preservation of transition-related information in the low-frequency modal space across 35 protein pairs. (**A**) Cumulative overlap at K=10 for linear Cα-ANM and BA-constrained modes; each point is one protein pair and the dashed line indicates identity. (**B**) Pair-median constrained-minus-linear cumulative overlap difference across K=1−50; shading is the simultaneous 95% protein-pair bootstrap band, and the open point marks K=10. (**C**,**D**) Cutoff sensitivity of the K=10 cumulative overlap difference and leading-mode concentration, respectively. Light points are individual protein-pair effects; diamonds and error bars are medians with BCa 95% confidence intervals.

At K=10, the constrained basis produced a higher cumulative overlap in 31 of 35 protein pairs. The pair-median constrained-minus-linear difference was +0.00952 (BCa 95% CI, +0.00702 to +0.02123; Figure 1A). Across K=1−50, the median difference remained positive through most of the low-frequency range, and the simultaneous bootstrap band showed that the advantage was not confined to a single choice of K (Figure 1B). Cumulative overlap decreased in four pairs; three decreases were small, whereas DnaK showed the largest decrease (−0.0357; Table A2).

**Figure A1:**
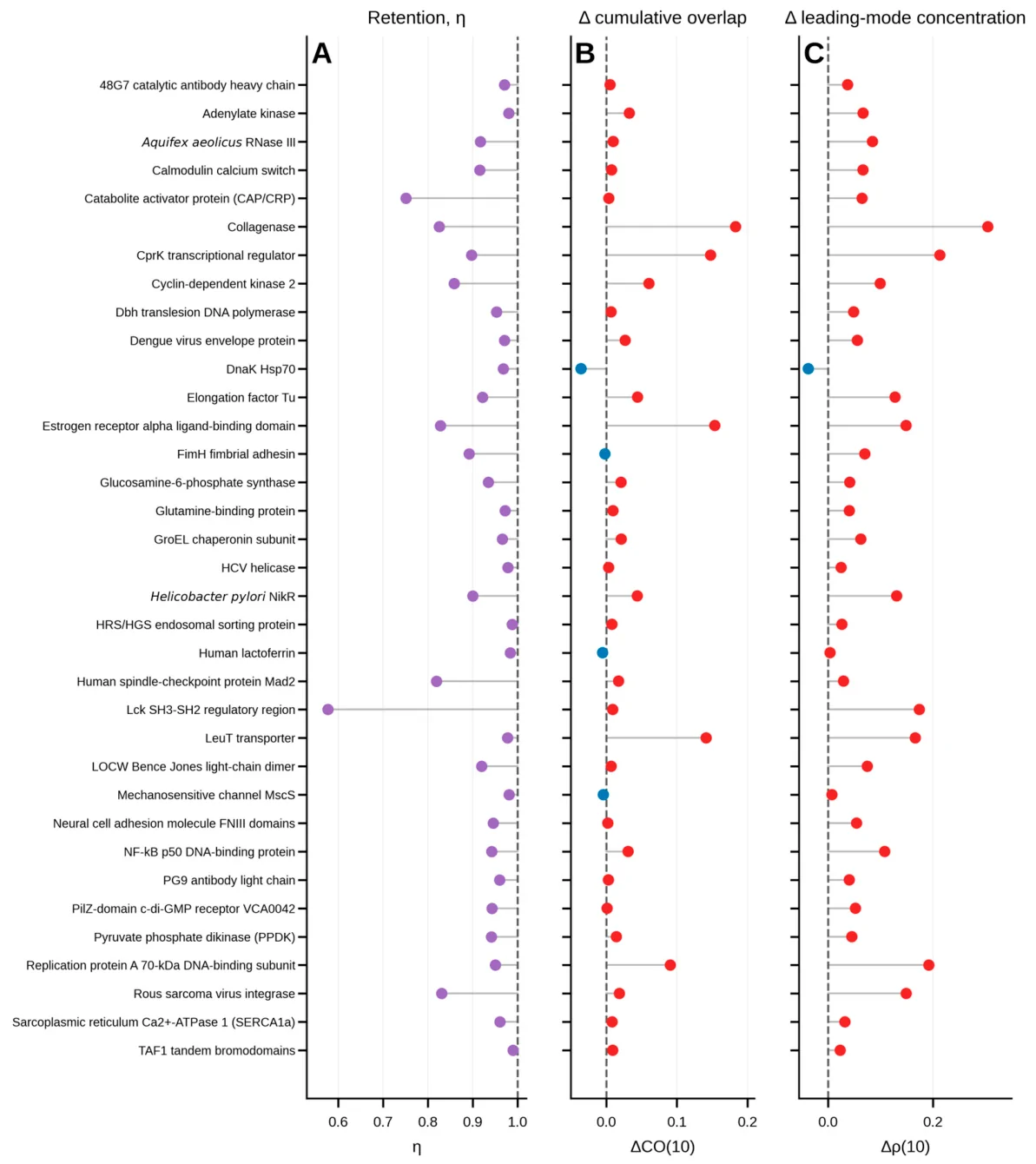
Constraint-space retention and leading-mode concentration across the 35-pair benchmark. (**A**) Fraction η of the mapped experimental displacement retained by the complete constrained modal space; horizontal stems extend from η = 1.0, the linear Cα-ANM reference. (**B**) BA-constrained minus linear Cα-ANM cumulative overlap, $\Delta \mathrm{CO}\left(10\right)$, at K=10. (**C**) Change in leading-mode concentration, $\Delta \rho \left(10\right)$. Protein pairs are listed alphabetically; each plotted value is the median of the two input-state summaries, and positive values in (**B**,**C**) favor the constrained basis. Purple points represent constrained-space retention in (**A**). In (**B**,**C**), red and blue points represent non-negative and negative changes, respectively; gray stems connect each point to the corresponding reference line (η=1 in (**A**) and Δ=0 in (**B**,**C**)).

The constrained space retained a median fraction η = 0.943 of the experimental displacement (range, 0.577–0.990), compared with η = 1 for the complete rigid-body-free linear Cα-ANM space. Although η was below 1 in every pair, the constrained space occupied only about one-third of the unconstrained internal Cartesian space (Section 2.4), while cumulative overlap at K=10 increased in 31 of 35 pairs and leading-mode concentration $\rho_{10}$ increased in 34 of 35; the median change in $\rho_{10}$ was +0.0625. The retained component was therefore usually redistributed toward the leading constrained modes. At cutoffs of 12, 15, and 18 Å, the median changes in cumulative overlap at K=10 were +0.00558, +0.00952, and +0.01700, respectively, and leading-mode concentration remained favorable (Figure 1C,D). Protein-resolved values are provided in Figure A1 and Table A2.

### 3.2. BA Constraints and ARAP Make Distinct Contributions to Local Cα Geometry

The modal-space analysis established that the constrained basis retained useful collective motion, but it did not identify which part of the method controlled each aspect of local geometry. We therefore compared four matched variants across all 35 pairs at actual Cα displacements of 1, 2, 3, 5, 8, and 10 Å. Matching the final displacement, rather than only the requested amplitude, prevented a method from appearing geometrically favorable simply because it moved the structure less.

**Figure 2:**
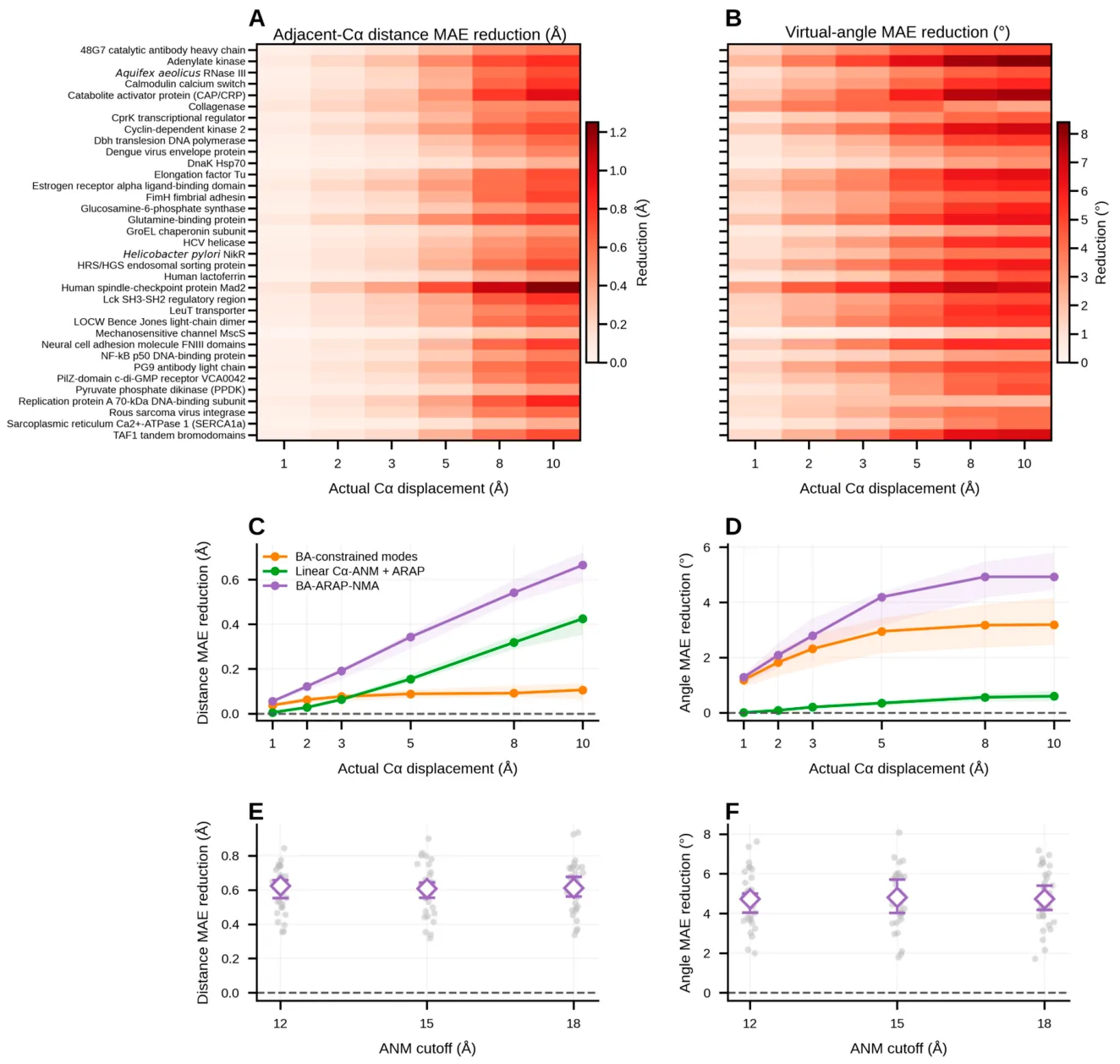
Local Cα geometry, four-way ablation, and cutoff robustness across the 35-pair benchmark. (**A**,**B**) Protein-resolved reduction in adjacent-Cα distance MAE and virtual-angle MAE for BA-ARAP-NMA relative to linear Cα-ANM at matched actual displacement; all values are positive, and darker red indicates a larger reduction. (**C**,**D**) Pair-median reduction for BA-constrained modes, linear Cα-ANM + ARAP, and BA-ARAP-NMA relative to linear Cα-ANM; ribbons show BCa 95% confidence intervals. (**E**,**F**) BA-ARAP-NMA effects at a requested amplitude of 10 Å across ANM cutoffs of 12, 15, and 18 Å. Light points are individual protein-pair effects; diamonds and error bars are medians with BCa 95% confidence intervals.

**Figure A2:**
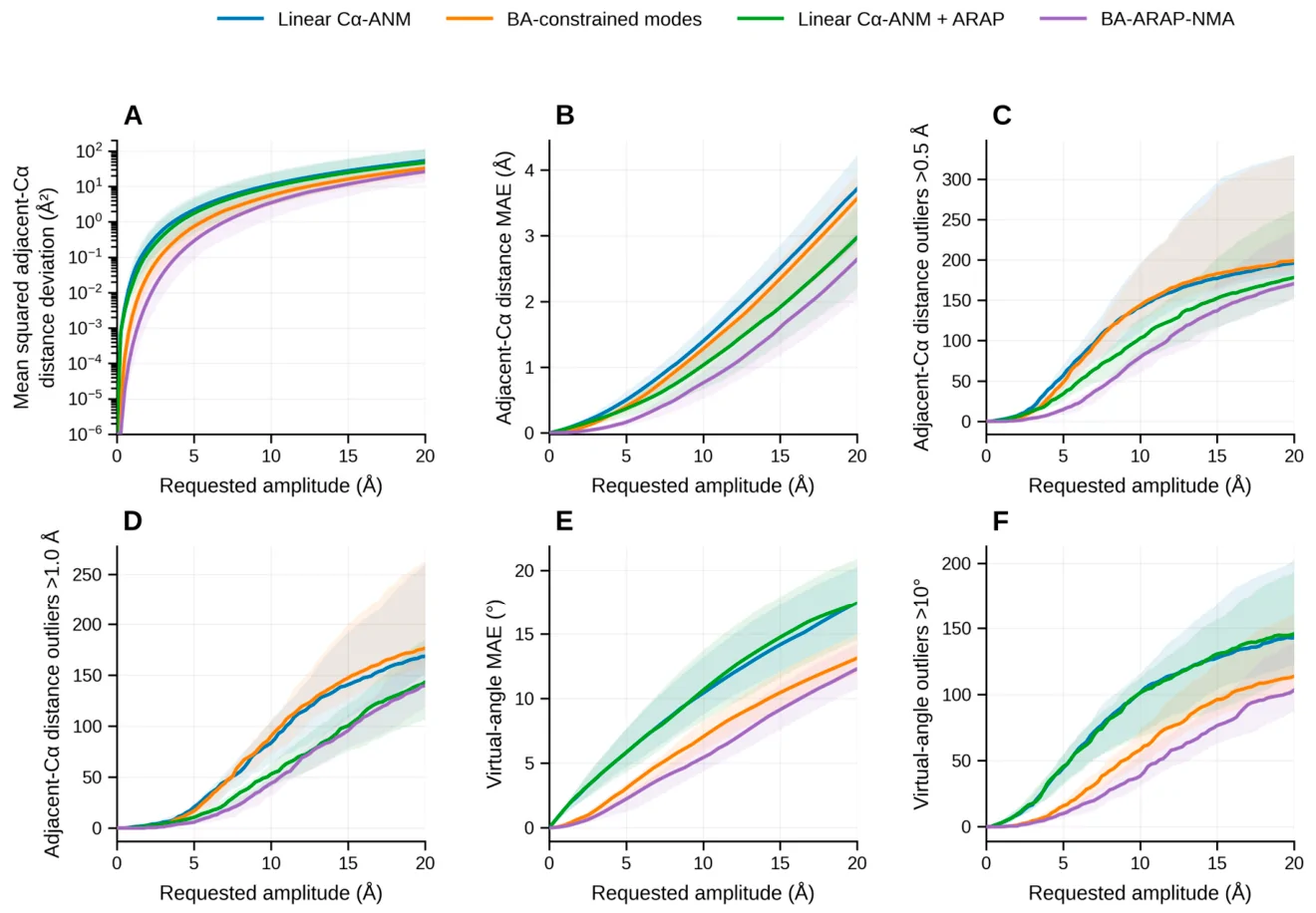
Pair-level local geometry profiles across the complete requested-amplitude range. Distance-based endpoints are shown in (**A**–**D**) and angle-based endpoints in (**E**,**F**). At each amplitude, each protein pair was summarized over the 20 branches defined by mode rank and orientation within each input state and then over the two input states. Curves show medians across 35 pairs, and ribbons show interquartile ranges.

Across the 35 pairs, the complete method accumulated substantially less adjacent-Cα distance and virtual-angle error than Linear Cα-ANM as actual displacement increased (Figure 2C,D). The protein-resolved heatmaps showed lower errors in every pair at every tested displacement (Figure 2A,B). At all six displacements, the exact sign tests remained significant after Holm correction for both local geometry endpoints (adjusted *p* = 3.49 × 10^−10^). Full requested-amplitude profiles are shown descriptively in Figure A2, complete method estimates are listed in Table A3, and component-level ablation estimates are listed in Table A4.

The four-way ablation showed that BA-constrained modes contributed strongly to virtual-angle control, whereas ARAP supplied most of the finite-amplitude adjacent-distance correction (Figure 2C,D; Table A4). At 10 Å of actual displacement, BA-constrained modes alone reduced the pair-median virtual-angle MAE by 3.19°, whereas the complete method reduced it by 4.92°; the complete method also reduced adjacent-distance MAE by 0.665 Å relative to linear Cα-ANM. In the requested-amplitude profiles (Figure A2), linear Cα-ANM + ARAP reached a median actual displacement of 10.39 Å at a requested amplitude of 10 Å, compared with 10.00 Å for linear Cα-ANM (Supplementary File S1). Its angle-error ordering at equal requested amplitude can therefore differ from the matched-actual-displacement result in Figure 2D. The cutoff analysis remained favorable at 12, 15, and 18 Å (Figure 2E,F; Table A8). At 10 Å actual displacement, the pair-median RMSD reduction was 0.003 Å for BA-constrained modes alone (BCa 95% CI, −0.002 to 0.012 Å), 0.398 Å for linear Cα-ANM + ARAP (0.243–0.604 Å), and 0.527 Å for the complete method (0.335–0.663 Å). Thus, at this displacement, ARAP made the larger independent contribution to paired-state RMSD, whereas the BA constraints made their clearest independent contribution to virtual-angle compatibility (Supplementary File S1).

### 3.3. Local Geometry Control Improves Generated-Structure Sets at the Protein-Pair Level

**Figure 3:**
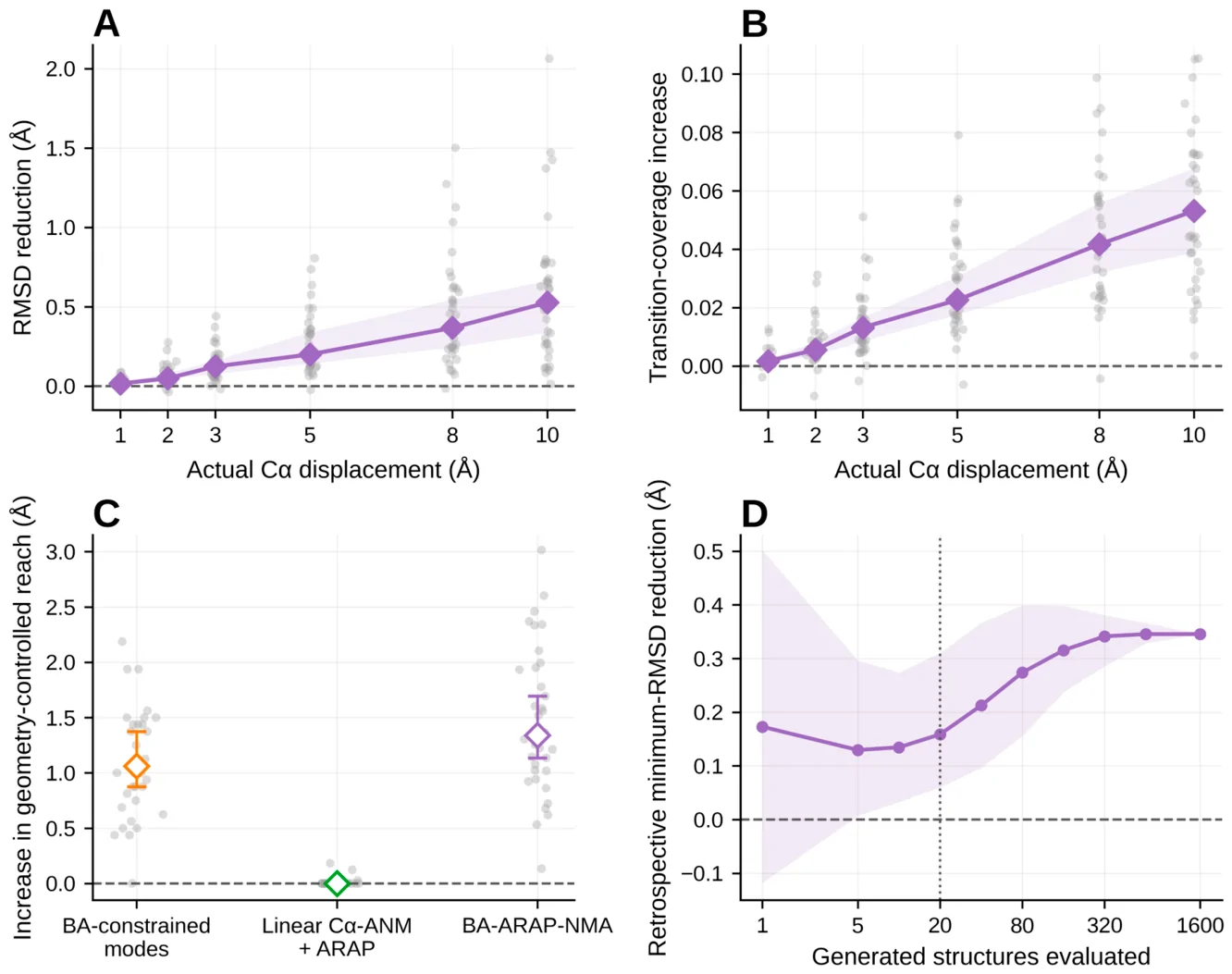
Protein-pair-level quality of the generated-structure sets. (**A**) Reduction in RMSD to the paired experimental structure at matched actual displacement. (**B**) Increase in transition coverage. Light points in A and B are individual protein-pair effects; diamonds and shaded ribbons show medians with BCa 95% confidence intervals. Input-state-specific estimates and order-invariant starting-structure sensitivity are reported in Supplementary File S1 and Table A5. (**C**) Increase in geometry-controlled reach for BA-constrained modes, linear Cα-ANM + ARAP, and BA-ARAP-NMA relative to linear Cα-ANM; light points are pair effects and diamonds with error bars are medians with BCa 95% confidence intervals. The linear Cα-ANM + ARAP median is 0.000 Å on the 0.25 Å grid. (**D**) Reduction in retrospective minimum RMSD as the number of generated structures evaluated increases. The vertical reference marks the 20-structure budget; shading is the 2.5–97.5% paired structure subset resampling range.

Improved local geometry would have limited practical value if the generated structure sets no longer approached the paired experimental state. At matched actual displacements from 1 to 10 Å, the overall within-pair effects favored BA-ARAP-NMA for RMSD to the paired structure and its normalized transition coverage representation (Figure 3A,B). Every BCa interval lay on the favorable side of zero, 32–35 of the 35 pairs favored BA-ARAP-NMA depending on displacement, and the exact sign tests remained significant after Holm correction. The two input-state effects were also compared within each pair without imposing a common direction across the heterogeneous transitions. The median absolute input-state difference in RMSD reduction increased from 0.032 Å at 1 Å displacement to 0.609 Å at 10 Å, indicating greater starting-structure sensitivity at larger deformations; the corresponding normalized coverage differences are reported in Table A5. Input-state-specific estimates are provided in Supplementary File S1.

Repeating the aggregation with matching tolerances of 0.10, 0.15, 0.20, and 0.25 Å retained all 35 protein pairs and both input states. Local geometry effects remained favorable for every pair, while paired-state effects favored BA-ARAP-NMA in 31 to 35 pairs, depending on displacement (Table A12). At the branch level, 97.6–99.9% of available branches favored BA-ARAP-NMA for local geometry. The corresponding proportion for paired-state RMSD and transition coverage was 60.4–69.7%. All 24 intervals from the hierarchical sensitivity analysis at the branch and protein-pair levels remained on the favorable side of zero (Table A13). Full tolerance and branch-level records are provided in Supplementary File S1.

We also measured the largest displacement reached before any adjacent-Cα distance outlier >1 Å or virtual-angle outlier >10° appeared. BA-ARAP-NMA increased this geometry-controlled reach by a median 1.340 Å relative to linear Cα-ANM (BCa 95% CI, 1.136–1.694 Å), with all 35 pairs favorable (Figure 3C; Table A6). BA-constrained modes alone contributed a median 1.063 Å. Linear Cα-ANM + ARAP had a median effect of 0.000 Å on the discrete 0.25 Å grid, with a BCa interval of 0–0.000089 Å.

The retrospective minimum-RMSD analysis provides an upper-bound description of the candidate space. With equal 1600-structure search budgets, BA-ARAP-NMA lowered the median minimum RMSD by 0.346 Å and increased transition coverage by 0.041, with 30 of 35 pairs favorable. Five pairs, including PPDK and MscS, did not show a lower retrospective minimum RMSD despite retaining the local geometry advantage. Fixed structure number analyses showed that the median RMSD reduction became more stable as the number of evaluated structures increased (Figure 3D; Table A7). The shaded region is the 2.5–97.5% range across paired structure subset resamples, not a confidence interval for a population of proteins; the vertical reference identifies the primary 20-structure budget.

### 3.4. HCV Helicase Illustrates Structure-Level Effects

**Figure 4:**
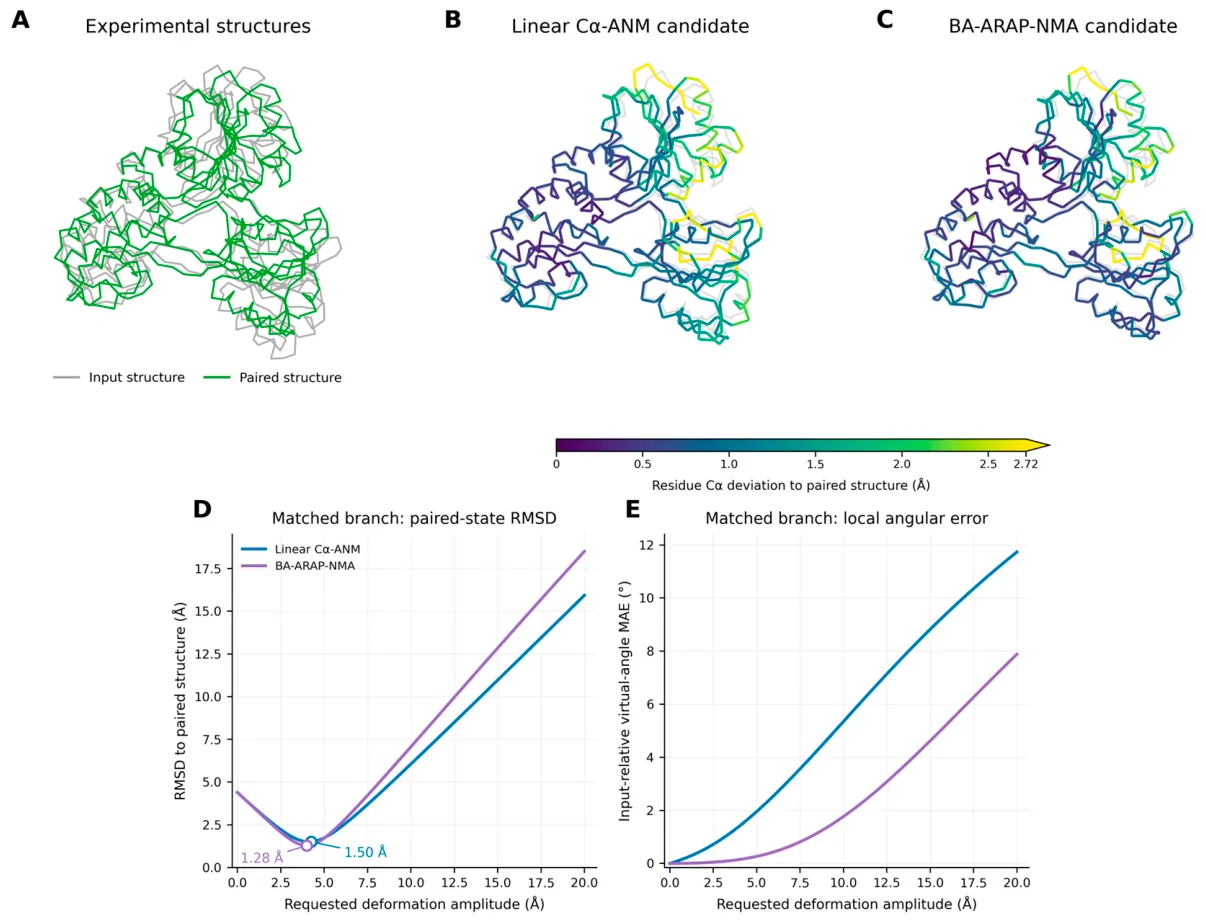
HCV helicase structure-level example using the common mapped helicase-domain Cα residues. (**A**) Superposition of the experimental input and paired structures. (**B**,**C**) Lowest-RMSD linear Cα-ANM and BA-ARAP-NMA candidates, colored by residue-level Cα deviation from the paired structure after global alignment. The two panels use a shared scale capped at the joint 95th percentile (2.72 Å); larger deviations are saturated at the highest color. (**D**) RMSD to the paired structure across the complete requested-amplitude grid; the minima are marked and labeled. (**E**) Input-relative virtual-angle MAE over the same grid. Source data are provided in Supplementary File S1.

HCV helicase illustrates how the benchmark-wide effects appear within one mode branch. The input and paired experimental structures differ by a collective rearrangement of the common helicase-domain Cα trace (Figure 4A). Linear Cα-ANM and BA-ARAP-NMA were compared at the same mode rank, orientation, and requested-amplitude grid (Figure 4B,C).

The lowest-RMSD linear Cα-ANM candidate had an RMSD of 1.50 Å and transition coverage of 0.658, whereas the BA-ARAP-NMA candidate reached 1.28 Å and 0.709. RMSD decreased and then increased with requested amplitude (Figure 4D), showing that amplitude indexes an ordered set of generated structures rather than a physical time trajectory.

At the selected candidates, BA-ARAP-NMA reduced the input-relative virtual-angle MAE from 1.53° to 0.15° and eliminated the six >10° virtual-angle outliers observed after linear scaling (Figure 4E).

### 3.5. Target-Free Comparison with NOLB Under Matched Displacement and Equal Structure Numbers

**Figure 5:**
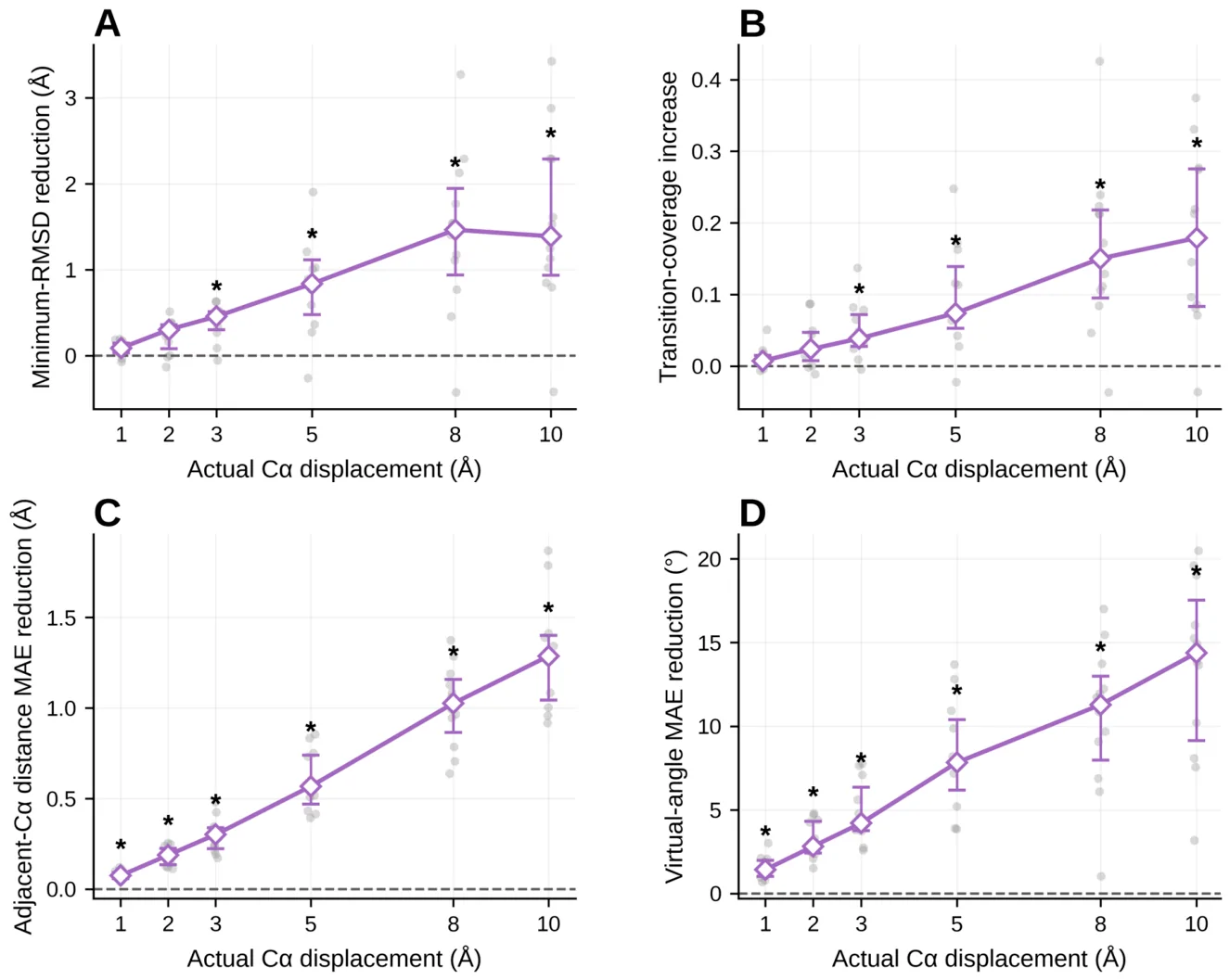
Target-free comparison with NOLB at matched actual displacement using 20 structures per method. Positive values favor BA-ARAP-NMA: NOLB minus BA-ARAP-NMA for minimum RMSD and local-error metrics, and BA-ARAP-NMA minus NOLB for transition coverage. (**A**) Minimum-RMSD reduction. (**B**) Transition Coverage increase. (**C**) Adjacent-Cα distance-MAE reduction for the minimum-RMSD candidate. (**D**) Virtual-angle-MAE reduction for the same candidate. Light points show individual protein-pair effects; diamonds and error bars show medians and BCa 95% confidence intervals. Asterisks indicate Holm-adjusted exact sign-test *p* < 0.05.

To compare BA-ARAP-NMA with an established nonlinear rigid-block method under comparable information access, matched actual displacement, and equal structure numbers, NOLB and BA-ARAP-NMA generated structures without access to the paired experimental state. Candidate pools were matched by actual common-Cα displacement, and the primary analysis evaluated equal sets of 20 structures. The subset spans all seven occupied size-by-RMSD groups and 12 distinct motion classes (Table A9). Within each fixed 20-structure set, the retrospectively selected minimum-RMSD BA-ARAP-NMA candidate had lower adjacent-Cα distance and virtual-angle errors than the corresponding NOLB candidate in every pair from 1 to 10 Å (Figure 5C,D). The comparator systems are listed in Table A9, and complete estimates are given in Table A10.

The minimum RMSD within each fixed 20-structure set favored BA-ARAP-NMA throughout the sampled range (Figure 5A), with transition coverage showing the corresponding positive effect (Figure 5B). All BCa intervals lay on the favorable side of zero. Directional consistency across protein pairs reached Holm-adjusted significance from 3 Å onward; at 1 and 2 Å, 10 of 12 pairs favored BA-ARAP-NMA, but the adjusted exact sign-test *p* value was 0.0771. The two NOLB-favoring pairs at these smaller displacements were PPDK and neural cell adhesion molecule FNIII domains; from 3 to 10 Å, only PPDK remained NOLB-favoring for minimum RMSD. Thus, the local geometry advantage was uniform, whereas paired-state candidate quality showed protein-level heterogeneity.

Candidate-budget analyses using 1, 5, and 10 structures retained lower local geometry error for BA-ARAP-NMA in all 12 pairs. In the primary 20-structure analysis, the overall protein-pair median remained favorable in 99.95–100% of the 2000 candidate-set resamples across all four endpoints and six displacements (Supplementary File S1).

### 3.6. Computational Cost

Across the 70 input-state calculations, median end-to-end time was 14.6 s for linear Cα-ANM, 14.2 s for BA-constrained modes, 48.0 s for linear Cα-ANM + ARAP, and 47.0 s for BA-ARAP-NMA. Because the four ablation variants were generated within one process per input-state calculation, peak resident memory was measured for the complete four-way ablation calculation rather than separately for each variant (median 94.7 MB; range, 75.3–512.2 MB). The 24 NOLB input-state calculations required a median 151.8 s (range, 103.1–828.3 s) and a median peak process-tree memory of 22.1 MB (range, 17.4–96.0 MB). Table A11 summarizes these end-to-end measurements.

## 4. Discussion

Across the 35-pair benchmark, the local Cα geometry benefit was reproduced in enzymes and nucleic-acid-associated proteins. It was also observed in chaperones and regulatory proteins. The remaining systems included transporters, a fold-switching protein, and MscS. Distance and virtual-angle errors were lower in every pair at all matched displacements, and the effect remained at 12, 15, and 18 Å cutoffs. This consistency is stronger than the paired-state result, which is more sensitive to the starting structure and to how well its low-frequency modes span the particular experimental displacement.

The central result of this study is not simply that BA-ARAP-NMA produced structures with better local geometry. It did so while retaining the transition-related collective information carried by the low-frequency modes. The local Cα geometry deteriorated progressively when linear Cα-ANM modes were enlarged by direct scaling. BA-ARAP-NMA consistently limited this deterioration across the sampled displacements, and the same pattern was evident in both distance- and angle-based measurements. At the same time, the constrained modes retained useful two-state information, and the complete method improved the paired-state candidate set at the protein-pair level. Local geometric control therefore did not improve the structures by merely keeping them close to the input conformation. The sampled low-frequency motions remained capable of producing substantial structural change, but that change was expressed through a more stable local Cα backbone. The local geometry measurements establish that the intended geometric control was achieved, whereas cumulative overlap and paired-state RMSD, with transition coverage as its normalized representation, show that this control did not eliminate useful collective motion. The NOLB comparison provides an external reference under matched information, displacement, and candidate-number conditions. Together, these results indicate that stronger local geometric control was obtained without discarding transition-related motion.

The ablation analysis also clarifies why both stages of the method are retained. The BA constraints reshape the initial modal basis and make their largest independent contribution to virtual-angle compatibility. Each virtual angle is determined by two adjacent and one next-nearest Cα distance, and all three have zero first-order change along a constrained mode. The linear-in-amplitude angular-drift term therefore vanishes near the input, leaving higher-order terms to dominate at finite amplitude. ARAP acts later, when a mode is extended to finite amplitude, and supplies most of the adjacent-distance correction. Their effects are complementary rather than interchangeable.

At first sight, the modal-space result may seem counterintuitive. Additional constraints reduce the admissible displacement space, yet cumulative overlap with the experimental two-state displacement was maintained or increased over the low-frequency ranges examined. The reason lies in how the constrained modes are obtained. BA-ARAP-NMA does not calculate a standard linear Cα-ANM basis and then discard directions that violate the local conditions. The local-distance and rigid-body constraints are applied before diagonalization; the Hessian is projected into the resulting admissible space and solved again. Consequently, both the directions and the ordering of the low-frequency modes are determined anew. Most of the experimental displacement remained representable after this recalculation, while a larger fraction of the retained component was concentrated in the leading constrained modes. A finite set of those modes could therefore attain the same or a higher cumulative overlap even though the complete constrained space retained somewhat less total displacement in some proteins. The constraints did not create conformational information that was absent from the elastic network. They changed how the information already present in that network was distributed among the recalculated low-frequency motions [7,8]. The constrained nullity was about one-third of the unconstrained internal Cartesian dimension across the benchmark. Retaining a median η of 0.943 in this smaller space indicates that most of the experimental displacement lies in locally compatible directions. The remaining dimension is of the same order as the internal torsional degrees of freedom of a Cα trace, placing the construction between unrestricted Cartesian NMA and a strictly internal-coordinate representation while retaining Cartesian output.

The geometric meaning of this redistribution is equally important. The local conditions keep adjacent and next-nearest Cα distances unchanged only to first order; they suppress local stretching, but they do not prescribe a hinge axis, a domain trajectory, or any other global displacement. Large-scale movement can still emerge through coordinated changes in local orientation and relative domain position. Internal-coordinate NMA provides a related precedent, because bond-length and bond-angle restrictions can preserve collective Cartesian motion and, in some cases, improve the representation of experimental conformational changes [10,11]. BA-ARAP-NMA extends this principle by separating the infinitesimal and finite-amplitude parts of structure generation. The constrained eigenproblem determines which low-frequency motions are locally compatible near the input structure. ARAP then acts on the coordinates produced by scaling one of those modes, accommodating finite local rotations while limiting the additional geometric drift that would otherwise accumulate. Neither component replaces the other.

BA-ARAP-NMA exposes the amplitude dependence of every selected mode rank and orientation instead of returning only one corrected endpoint. The multi-model Cα files and frame-level measurements show when a collective movement begins to incur local geometric damage and allow alternative branches to be compared. In applications with one experimental structure, generated structures can be assessed using local geometry, structural context, or independent experimental information before downstream rebuilding and refinement.

The external comparison focused on NOLB because its nonlinear Cartesian structure-generation framework allowed the two methods to be evaluated under matched information access, actual displacement, and candidate-set size.

Under target-free random-ensemble generation, the methods were compared at matched actual displacement using equal sets of 20 structures. The minimum-RMSD candidate selected retrospectively from each BA-ARAP-NMA set had lower local Cα errors than the corresponding NOLB candidate. BA-ARAP-NMA also had a more favorable median set-level minimum RMSD. PPDK remained a counterexample for minimum RMSD. The comparison represents one controlled NOLB protocol; different ensemble sizes, guidance strategies, or downstream objectives may yield different trade-offs.

Collagenase and PPDK reveal a different limitation, one that is already present in the underlying single-mode NMA description. In matched-actual-displacement comparisons, BA-ARAP-NMA improved pair-level RMSD and its normalized transition coverage in both proteins without fully recovering the experimental endpoint difference. PPDK, however, did not retain a lower retrospective minimum RMSD when the complete 1600-structure candidate sets from both input states were summarized. The structural changes involved are not simple enlargements of one uniform collective displacement. Calcium binding in the collagenase pair reorganizes the N-terminal linker from an α-helical segment into a β-strand anchored to the collagen-binding domain [20]. PPDK undergoes a much larger rearrangement in which the central phosphohistidine domain swivels by approximately 111° between catalytic centers separated by about 45 Å, together with an opening of the nucleotide-binding cleft [33]. A local secondary-structure conversion and a strongly coupled domain movement may each draw on several low-frequency directions, and neither need be represented completely by a single mode calculated from one endpoint.

The comparison between cumulative overlap and structure generation supports this view. When the first ten constrained modes were considered together, cumulative overlap increased in both collagenase and PPDK. The structure-generation benchmark, however, sampled those modes separately and selected the best structure obtained from one mode rank and orientation. Information distributed across several low-frequency directions can therefore be present in the modal space without appearing fully in any single generated branch. Collagenase illustrates this distinction especially clearly. It showed the largest increase in the fraction of retained displacement carried by the first ten constrained modes, yet it also retained the smallest fraction of the complete experimental displacement and exhibited the largest first-order local-distance change (Supplementary File S1). The constrained calculation represented the collective component more efficiently, but the linker reorganization remained difficult to reproduce through one mode. The limited absolute coverage in these cases is therefore more consistent with the expressive range of single-mode NMA than with a loss introduced by the local constraints.

The same observation suggests how the method can develop further. When a conformational change is distributed across several collective directions, a small set of constrained modes could be allowed to contribute jointly to one generated structure before ARAP correction. A different extension may be needed when a global movement is accompanied by localized remodeling. In a case such as the collagenase linker, the mode-based Cα movement could be followed by a limited local-relaxation or restraint-guided step, while the remainder of the structure remains under the existing geometric control. The present implementation also remains a Cα-level method. Subsequent rebuilding must introduce side-chain packing and full-atom stereochemistry, including solvent-mediated interactions and hydrogen bonding [34,35]. At the largest amplitudes, smoothly varying non-local steric restraints may also help prevent excessive compression between distant regions [9,15]. These extensions would broaden the structural range of BA-ARAP-NMA while preserving its defining features: recalculated constrained modes, finite-rotation ARAP correction, and amplitude-resolved models that can be inspected and compared.

The additional computational cost relative to linear Cα-ANM (Section 3.6) arose mainly from repeated finite-amplitude ARAP mapping and frame-level quality evaluation. The constrained modal calculation itself remained comparable in cost because the projected eigensystem was solved in a subspace with a median dimension of 235, approximately one-third of the unconstrained internal Cartesian dimension. The small timing difference between BA-constrained and linear modes is benchmark—and implementation—specific and is not presented as a general speed advantage. Calculations for different mode ranks and orientations are independent and can be parallelized. The present Python implementation prioritizes transparent, reproducible calculations over low-level performance optimization; complete calculation-level timing and memory records are provided in Supplementary File S1.

## 5. Conclusions

BA-ARAP-NMA was developed to address the progressive loss of local backbone geometry caused by directly enlarging Cα modes. By recalculating the low-frequency modes under first-order local-distance constraints and applying co-rotational ARAP, the method reduced local distortion while retaining most of the transition-related conformational information carried by the modal space. Across 35 experimentally determined structure pairs, the BA constraints and ARAP mapping made distinct contributions to local geometry. At matched actual displacement, the complete method reduced adjacent-Cα distance and virtual-angle errors in every pair. It also increased geometry-controlled reach and improved the paired-state candidate set at the protein-pair level. The constraints did not simply remove motions from the conventional Cα-ANM basis; they changed how the retained conformational information was distributed within the low-frequency space. The local geometric effects were robust to both input structures and to ANM cutoffs of 12, 15, and 18 Å. Under the controlled target-free NOLB protocol with equal structure numbers, the retrospectively selected minimum-RMSD BA-ARAP-NMA candidate had lower local Cα errors in all 12 comparator pairs. BA-ARAP-NMA also had a lower median set-level minimum RMSD across 1–10 Å, although PPDK remained a protein-level counterexample for paired-state RMSD. The present framework is most effective when structural change is dominated by collective low-frequency motion and is best viewed as an amplitude-resolved generator of inspectable Cα structures that can be prioritized with independent evidence and subsequently rebuilt or refined.

## Data Availability

The BA-ARAP-NMA v1.0.0 source code is available at https://github.com/chriszyu9605/BA-ARAP-NMA and https://github.com/chriszyu9605/BA-ARAP-NMA/releases/tag/v1.0.0 [30,31]. Supplementary File S1 provides the analysis scripts used to calculate the reported diagnostics; exact prepared inputs; numerical source data; benchmark selection, exclusion, and mapping records; statistical scripts; direct figure-generation scripts; NOLB comparison records; accepted common-Cα coordinates; computational-cost records; and reproducibility files. Experimental structures were obtained from the Protein Data Bank using the accession codes listed in Table A1. Both online resources were accessed on 20 August 2026.

## Supplementary Materials

The following supporting information can be downloaded at https://www.mdpi.com/article/10.3390/biology15171533/s1, The separate Supplementary File S1 provides the BA-ARAP-NMA v1.0.0 source code; the analysis scripts used to calculate the reported diagnostics, the 35-pair benchmark, prepared structures and residue mappings; pair-, input-state-, branch-, and frame-level source data; modal-space and cutoff analyses; the 12-pair NOLB records; candidate-set resampling results; accepted common-Cα coordinate arrays; statistical outputs; timing measurements; direct figure-generation scripts; and file integrity records. Within this archive, the software packages are designated Software S1 (BA-ARAP-NMA v1.0.0) and Software S2 (the companion local-distance diagnostic package).

## Appendix A

Appendix A provides the benchmark composition and modal-space diagnostics, followed by full-amplitude profiles and component- and protein-pair-level inference. It then reports starting structure sensitivity and geometry-controlled reach. Candidate number and cutoff robustness are followed by matched-displacement tolerance and branch-level robustness. The final section presents the target-free NOLB comparison and computational-cost summaries. Complete machine-readable data at the pair, input-state, branch, and frame levels are provided in Supplementary File S1.

### Appendix A.1. Benchmark Composition and Modal-Space Diagnostics

**Table A1:** Experimentally determined protein structure pairs in the 35-pair benchmark.

Protein System	Input Structure	Paired Second-State Structure	Common Cα	Pair RMSD (Å)	Structural Change Represented	Structural Reference(s)
48G7 catalytic antibody heavy chain	1GAF chain H	1AJ7 chain H	217	5.428	Immunoglobulin domain reorientation	[36,37]
Adenylate kinase	4AKE chain A	1AKE chain A	214	7.131	Enzyme lid closure	[38,39]
*Aquifex aeolicus* RNase III	1YYW chain A	2NUG chain A	216	11.877	Nucleic-acid-processing rearrangement	[40,41]
Calmodulin calcium switch	1CFD chain A	1CLL chain A	144	10.829	Calcium sensor rearrangement	[42,43]
Catabolite activator protein (CAP/CRP)	3FWE chain B	4HZF chain A	200	11.045	DNA binding domain rearrangement	[44,45]
Collagenase	1NQD chain A	1NQJ chain B	111	7.277	Linker hinge rearrangement	[20]
CprK transcriptional regulator †	2H6B chain B	3E6C chain C	225	15.843	Regulatory domain rearrangement	[46,47]
Cyclin-dependent kinase 2	1HCL chain A	1FIN chain A	294	3.971	Kinase activation	[48,49]
Dbh translesion DNA polymerase †	1K1Q chain B	4NLG chain A	341	5.873	Polymerase domain rearrangement	[50,51]
Dengue virus envelope protein	1OAN chain A	1OK8 chain A	380	11.837	Viral fusion rearrangement	[52,53]
DnaK Hsp70	2KHO chain A	4B9Q chain A	599	34.384	Hsp70 allosteric cycle	[54,55]
Elongation factor Tu	1TUI chain A	1EFT chain A	397	10.282	GTPase domain switch	[56,57]
Estrogen receptor alpha ligand-binding domain	3ERD chain A	3ERT chain A	244	4.809	Nuclear receptor helix 12 switch	[58]
FimH fimbrial adhesin	3RFZ chain D	3JWN chain H	279	14.620	Adhesin domain rearrangement	[59,60]
Glucosamine-6-phosphate synthase	4AMV chain A	3OOJ chain A	608	23.428	Large enzyme domain motion	[61,62]
Glutamine-binding protein	1GGG chain A	1WDN chain A	220	5.338	Binding protein closure	[63,64]
GroEL chaperonin subunit †	1OEL chain A	1AON chain A	524	12.378	Chaperonin domain rearrangement	[65,66]
HCV helicase	8OHM chain A	1CU1 chain A	435	4.395	Helicase rearrangement	[67,68]
*Helicobacter pylori* NikR	2WVD chain B	2WVF chain A	134	7.785	Metal-sensing regulatory rearrangement	[69]
HRS/HGS endosomal sorting protein	4AVX chain A	3ZYQ chain A	216	5.015	Endosomal-sorting interdomain motion	[70,71,72]
Human lactoferrin	1LCF chain A	1CB6 chain A	691	6.438	Transferrin lobe closure	[73,74]
Human spindle-checkpoint protein Mad2 †	1DUJ chain A	1KLQ chain A	187	9.841	Topological fold switch	[75,76]
Lck SH3-SH2 regulatory region	4D8K chain A	1X27 chain D	163	10.617	Regulatory domain rearrangement	[77,78]
LeuT transporter †	3TT1 chain A	3TT3 chain A	492	3.627	Alternating access transport	[79]
LOCW Bence Jones light-chain dimer	4BJL chain B	1BJM chain A	215	13.024	Immunoglobulin domain reorientation	[80]
Mechanosensitive channel MscS †	7OO8 chain A	7OOA chain A	261	6.918	Mechanosensitive channel gating	[81]
Neural cell adhesion molecule FNIII domains †	2VKX chain B	2VKW chain A	196	8.610	Adhesion-domain reorientation	[82]
NF-kB p50 DNA-binding protein	1OOA chain A	1NFK chain A	312	5.382	DNA binding domain rearrangement	[83,84]
PG9 antibody light chain †	3MUH chain L	3U2S chain B	211	7.687	Immunoglobulin domain reorientation	[85,86]
PilZ-domain c-di-GMP receptor VCA0042	3KYG chain B	2RDE chain A	224	9.533	Second messenger receptor rearrangement	[87,88]
Pyruvate phosphate dikinase (PPDK) †	1KBL chain A	2R82 chain A	872	11.653	Large enzyme domain motion	[33,89]
Replication protein A 70-kDa DNA-binding subunit	1FGU chain A	1JMC chain A	238	8.303	DNA binding domain rearrangement	[90,91]
Rous sarcoma virus integrase †	4FW1 chain B	1C0M chain C	216	14.416	Integrase domain rearrangement	[92,93]
Sarcoplasmic reticulum Ca^2+^-ATPase 1 (SERCA1a) †	1SU4 chain A	1IWO chain A	994	13.971	P-type ATPase transport cycle	[94,95]
TAF1 tandem bromodomains †	3UV5 chain A	1EQF chain A	249	10.775	Tandem domain reorientation	[96,97]

† Pair included in the 12-pair NOLB comparator set selected before NOLB performance was examined. The order of the two structures is used only for reporting; each structure was separately used as an input. The HCV pair includes only the common helicase domain residues shared by 8OHM and 1CU1; NS3 protease domain residues present only in 1CU1 were excluded. Both Lck entries contain the human Lck SH3–SH2 regulatory region; the title of the p130Cas structural paper refers more broadly to Src family regulation. Complete residue mappings and structure provenance are provided in Supplementary File S1.

**Table A2:** Modal retention and leading-mode concentration by protein pair.

Protein System	Constrained Retention η	Δ Cumulative Overlap, K=10	Δ Leading-Mode Concentration, K=10
48G7 catalytic antibody heavy chain	0.971	+0.0055	+0.0374
Adenylate kinase	0.98	+0.0327	+0.0668
*Aquifex aeolicus* RNase III	0.917	+0.0098	+0.0847
Calmodulin calcium switch	0.916	+0.0076	+0.0666
Catabolite activator protein (CAP/CRP)	0.751	+0.0037	+0.0649
Collagenase	0.825	+0.183	+0.304
CprK transcriptional regulator	0.898	+0.148	+0.213
Cyclin-dependent kinase 2	0.859	+0.0605	+0.0995
Dbh translesion DNA polymerase	0.953	+0.0069	+0.0489
Dengue virus envelope protein	0.971	+0.0268	+0.056
DnaK Hsp70	0.968	−0.0357	−0.0376
Elongation factor Tu	0.922	+0.0443	+0.128
Estrogen receptor alpha ligand-binding domain	0.828	+0.154	+0.149
FimH fimbrial adhesin	0.892	−0.0019	+0.0702
Glucosamine-6-phosphate synthase	0.935	+0.021	+0.0415
Glutamine-binding protein	0.972	+0.0095	+0.0406
GroEL chaperonin subunit	0.967	+0.0212	+0.0625
HCV helicase	0.979	+0.0034	+0.025
*Helicobacter pylori* NikR	0.9	+0.0439	+0.131
HRS/HGS endosomal sorting protein	0.988	+0.0081	+0.0265
Human lactoferrin	0.984	−0.0052	+0.0039
Human spindle-checkpoint protein Mad2	0.819	+0.0174	+0.0296
Lck SH3-SH2 regulatory region	0.577	+0.0091	+0.174
LeuT transporter	0.978	+0.141	+0.166
LOCW Bence Jones light-chain dimer	0.92	+0.007	+0.0749
Mechanosensitive channel MscS	0.981	−0.0041	+0.0075
Neural cell adhesion molecule FNIII domains	0.946	+0.0022	+0.0544
NF-kB p50 DNA-binding protein	0.942	+0.031	+0.108
PG9 antibody light chain	0.96	+0.0031	+0.0405
PilZ-domain c-di-GMP receptor VCA0042	0.943	+0.0012	+0.0523
Pyruvate phosphate dikinase (PPDK)	0.942	+0.0143	+0.0454
Replication protein A 70-kDa DNA-binding subunit	0.95	+0.0907	+0.192
Rous sarcoma virus integrase	0.831	+0.0186	+0.149
Sarcoplasmic reticulum Ca2+-ATPase 1 (SERCA1a)	0.961	+0.0083	+0.0323
TAF1 tandem bromodomains	0.99	+0.0091	+0.0232

Each protein-pair value is the median of the two input-state summaries. η is the fraction of the mapped experimental displacement representable by the complete constrained modal space. Δ values are BA-constrained modes minus linear Cα-ANM. Positive Δ cumulative overlap indicates better representation by the first ten modes; positive Δ leading-mode concentration indicates that a larger fraction of the retained component is concentrated in those modes. Pair- and input-state-level first-order local-distance-change values are provided in Supplementary File S1.

### Appendix A.3. Component Contributions and Protein-Pair-Level Inference

**Table A3:** Complete-method effects relative to linear Cα-ANM at matched actual displacement.

Endpoint	Displacement (Å)	Median Improvement	BCa 95% CI	Pairs Favorable	Holm *p*
Adjacent-Cα distance MAE	1	0.0548	0.0465 to 0.0616	35/35	3.49 × 10^−10^
RMSD to paired structure	1	0.0158	0.0126 to 0.0238	32/35	4.18 × 10^−7^
Transition coverage	1	0.00169	0.00121 to 0.00228	32/35	4.18 × 10^−7^
Virtual-angle MAE	1	1.28	1.05 to 1.4	35/35	3.49 × 10^−10^
Adjacent-Cα distance MAE	2	0.122	0.107 to 0.127	35/35	3.49 × 10^−10^
RMSD to paired structure	2	0.0483	0.0451 to 0.0797	33/35	7.35 × 10^−8^
Transition coverage	2	0.00557	0.00373 to 0.00834	33/35	7.35 × 10^−8^
Virtual-angle MAE	2	2.08	1.63 to 2.53	35/35	3.49 × 10^−10^
Adjacent-Cα distance MAE	3	0.191	0.154 to 0.203	35/35	3.49 × 10^−10^
RMSD to paired structure	3	0.123	0.077 to 0.162	34/35	1.05 × 10^−8^
Transition coverage	3	0.0132	0.00843 to 0.0167	34/35	1.05 × 10^−8^
Virtual-angle MAE	3	2.79	2.34 to 3.44	35/35	3.49 × 10^−10^
Adjacent-Cα distance MAE	5	0.343	0.291 to 0.361	35/35	3.49 × 10^−10^
RMSD to paired structure	5	0.199	0.142 to 0.338	34/35	1.05 × 10^−8^
Transition coverage	5	0.0226	0.0176 to 0.0307	34/35	1.05 × 10^−8^
Virtual-angle MAE	5	4.19	3.16 to 4.38	35/35	3.49 × 10^−10^
Adjacent-Cα distance MAE	8	0.541	0.501 to 0.599	35/35	3.49 × 10^−10^
RMSD to paired structure	8	0.367	0.245 to 0.545	34/35	1.05 × 10^−8^
Transition coverage	8	0.0417	0.0321 to 0.0559	34/35	1.05 × 10^−8^
Virtual-angle MAE	8	4.92	4.17 to 5.48	35/35	3.49 × 10^−10^
Adjacent-Cα distance MAE	10	0.665	0.59 to 0.721	35/35	3.49 × 10^−10^
RMSD to paired structure	10	0.527	0.335 to 0.663	35/35	3.49 × 10^−10^
Transition coverage	10	0.0531	0.0388 to 0.0677	35/35	3.49 × 10^−10^
Virtual-angle MAE	10	4.92	4.46 to 5.81	35/35	3.49 × 10^−10^

Positive values favor complete BA-ARAP-NMA. For adjacent-distance MAE, virtual-angle MAE, and RMSD, improvement is defined as linear Cα-ANM minus BA-ARAP-NMA. For transition coverage, improvement is defined as BA-ARAP-NMA minus linear Cα-ANM. The protein pair is the independent statistical unit. Wilcoxon signed-rank tests, Hodges–Lehmann shifts, and rank-biserial effects are provided in Supplementary File S1.

**Table A4:** Four-variant ablation effects on local Cα geometry at matched actual displacement.

Variant Comparison	Endpoint	Displacement (Å)	Median Improvement [BCa 95% CI]	Pairs Favorable	Holm *p*
BA-constrained modes vs. linear Cα-ANM	Adjacent-Cα distance MAE	1	0.0373 [0.033, 0.0436]	35/35	3.49 × 10^−10^
BA-constrained modes vs. linear Cα-ANM	Adjacent-Cα distance MAE	2	0.0622 [0.0504, 0.0734]	35/35	3.49 × 10^−10^
BA-constrained modes vs. linear Cα-ANM	Adjacent-Cα distance MAE	3	0.0765 [0.0556, 0.0856]	35/35	3.49 × 10^−10^
BA-constrained modes vs. linear Cα-ANM	Adjacent-Cα distance MAE	5	0.0884 [0.0699, 0.107]	34/35	6.29 × 10^−9^
BA-constrained modes vs. linear Cα-ANM	Adjacent-Cα distance MAE	8	0.0916 [0.0763, 0.127]	33/35	7.35 × 10^−8^
BA-constrained modes vs. linear Cα-ANM	Adjacent-Cα distance MAE	10	0.106 [0.0627, 0.137]	32/35	4.18 × 10^−7^
BA-constrained modes vs. linear Cα-ANM	Virtual-angle MAE	1	1.18 [0.935, 1.37]	35/35	3.49 × 10^−10^
BA-constrained modes vs. linear Cα-ANM	Virtual-angle MAE	2	1.82 [1.34, 2.25]	35/35	3.49 × 10^−10^
BA-constrained modes vs. linear Cα-ANM	Virtual-angle MAE	3	2.31 [1.65, 2.84]	35/35	3.49 × 10^−10^
BA-constrained modes vs. linear Cα-ANM	Virtual-angle MAE	5	2.95 [2.16, 3.44]	35/35	3.49 × 10^−10^
BA-constrained modes vs. linear Cα-ANM	Virtual-angle MAE	8	3.17 [2.37, 3.93]	35/35	3.49 × 10^−10^
BA-constrained modes vs. linear Cα-ANM	Virtual-angle MAE	10	3.19 [2.46, 4.17]	34/35	2.09 × 10^−9^
Linear Cα-ANM + ARAP vs. linear Cα-ANM	Adjacent-Cα distance MAE	1	0.00526 [0.00413, 0.00688]	35/35	3.49 × 10^−10^
Linear Cα-ANM + ARAP vs. linear Cα-ANM	Adjacent-Cα distance MAE	2	0.0276 [0.0209, 0.0314]	35/35	3.49 × 10^−10^
Linear Cα-ANM + ARAP vs. Linear Cα-ANM	Adjacent-Cα distance MAE	3	0.0632 [0.0534, 0.0715]	35/35	3.49 × 10^−10^
Linear Cα-ANM + ARAP vs. linear Cα-ANM	Adjacent-Cα distance MAE	5	0.155 [0.141, 0.177]	35/35	3.49 × 10^−10^
Linear Cα-ANM + ARAP vs. linear Cα-ANM	Adjacent-Cα distance MAE	8	0.318 [0.289, 0.35]	35/35	3.49 × 10^−10^
Linear Cα-ANM + ARAP vs. linear Cα-ANM	Adjacent-Cα distance MAE	10	0.425 [0.354, 0.449]	35/35	3.49 × 10^−10^
Linear Cα-ANM + ARAP vs. linear Cα-ANM	Virtual-angle MAE	1	0.00475 [−9.45 × 10^−4^, 0.00898]	23/35	0.0895
Linear Cα-ANM + ARAP vs. linear Cα-ANM	Virtual-angle MAE	2	0.0836 [0.0549, 0.098]	31/35	1.39 × 10^−5^
Linear Cα-ANM + ARAP vs. linear Cα-ANM	Virtual-angle MAE	3	0.203 [0.123, 0.234]	32/35	2.51 × 10^−6^
Linear Cα-ANM + ARAP vs. linear Cα-ANM	Virtual-angle MAE	5	0.346 [0.265, 0.442]	32/35	2.51 × 10^−6^
Linear Cα-ANM + ARAP vs. linear Cα-ANM	Virtual-angle MAE	8	0.558 [0.414, 0.685]	28/35	0.00153
Linear Cα-ANM + ARAP vs. linear Cα-ANM	Virtual-angle MAE	10	0.598 [0.413, 0.805]	27/35	0.00376
BA-ARAP-NMA vs. linear Cα-ANM	Adjacent-Cα distance MAE	1	0.0548 [0.0465, 0.0616]	35/35	3.49 × 10^−10^
BA-ARAP-NMA vs. linear Cα-ANM	Adjacent-Cα distance MAE	2	0.122 [0.107, 0.127]	35/35	3.49 × 10^−10^
BA-ARAP-NMA vs. linear Cα-ANM	Adjacent-Cα distance MAE	3	0.191 [0.154, 0.203]	35/35	3.49 × 10^−10^
BA-ARAP-NMA vs. linear Cα-ANM	Adjacent-Cα distance MAE	5	0.343 [0.291, 0.361]	35/35	3.49 × 10^−10^
BA-ARAP-NMA vs. linear Cα-ANM	Adjacent-Cα distance MAE	8	0.541 [0.501, 0.599]	35/35	3.49 × 10^−10^
BA-ARAP-NMA vs. linear Cα-ANM	Adjacent-Cα distance MAE	10	0.665 [0.59, 0.721]	35/35	3.49 × 10^−10^
BA-ARAP-NMA vs. linear Cα-ANM	Virtual-angle MAE	1	1.28 [1.05, 1.4]	35/35	3.49 × 10^−10^
BA-ARAP-NMA vs. linear Cα-ANM	Virtual-angle MAE	2	2.08 [1.63, 2.53]	35/35	3.49 × 10^−10^
BA-ARAP-NMA vs. linear Cα-ANM	Virtual-angle MAE	3	2.79 [2.34, 3.44]	35/35	3.49 × 10^−10^
BA-ARAP-NMA vs. linear Cα-ANM	Virtual-angle MAE	5	4.19 [3.16, 4.38]	35/35	3.49 × 10^−10^
BA-ARAP-NMA vs. linear Cα-ANM	Virtual-angle MAE	8	4.92 [4.17, 5.48]	35/35	3.49 × 10^−10^
BA-ARAP-NMA vs. linear Cα-ANM	Virtual-angle MAE	10	4.92 [4.46, 5.81]	35/35	3.49 × 10^−10^

Positive values favor the named variant. The three comparisons against linear Cα-ANM correspond to the four matched variants shown in Figure 2C,D. Incremental full-versus-component contrasts and nonparametric sensitivity analyses are provided in Supplementary File S1.

**Table A5:** Starting-structure sensitivity in paired-state improvement.

Endpoint	Displacement (Å)	Median Absolute Input-State Difference	BCa 95% CI Low	BCa 95% CI High	Pairs
RMSD reduction (Å)	1	0.0321	0.00831	0.0425	35
Transition Coverage increase	1	0.00349	7.83 × 10^−4^	0.00563	35
RMSD reduction (Å)	2	0.103	0.0611	0.139	35
Transition Coverage increase	2	0.012	0.00834	0.0198	35
RMSD reduction (Å)	3	0.189	0.143	0.283	35
Transition Coverage increase	3	0.0263	0.018	0.0347	35
RMSD reduction (Å)	5	0.325	0.193	0.426	35
Transition Coverage increase	5	0.0432	0.0342	0.0536	35
RMSD reduction (Å)	8	0.516	0.398	0.698	35
Transition Coverage increase	8	0.0825	0.0436	0.095	35
RMSD reduction (Å)	10	0.609	0.458	0.869	35
Transition Coverage increase	10	0.0822	0.0523	0.104	35

Each value is the absolute difference between the two input-state effects within the same protein pair; medians and BCa 95% confidence intervals are calculated across 35 pairs. RMSD reduction is linear Cα-ANM minus BA-ARAP-NMA, whereas transition coverage increase is BA-ARAP-NMA minus linear Cα-ANM. Transition coverage is the within-pair normalized representation of RMSD reduction. Because absolute differences have no common direction, no directional sign test or Holm adjustment was applied. Input-state-specific values are provided in Supplementary File S1.

**Table A6:** Geometry-controlled reach before severe local Cα outliers appeared.

Variant Comparison	Median Increase [BCa 95% CI] (Å)	Pairs Favorable	Exact Sign *p*	Wilcoxon *p*	Hodges–Lehmann Shift; Rank-Biserial Effect
BA-constrained modes vs. linear Cα-ANM	1.06 [0.875, 1.38]	35/35	5.82 × 10^−11^	1.16 × 10^−10^	1.06; 1
Linear Cα-ANM + ARAP vs. linear Cα-ANM	0 [0, 8.87 × 10^−5^]	14/35	0.00418	7.63 × 10^−4^	9.8 × 10^−4^; 0.882
BA-ARAP-NMA vs. linear Cα-ANM	1.34 [1.14, 1.69]	35/35	5.82 × 10^−11^	5.82 × 10^−11^	1.44; 1

Positive values indicate that the named variant reached a larger actual displacement before either an adjacent-Cα distance outlier > 1 Å or a virtual-angle outlier > 10° appeared. Exact sign tests exclude zero pairwise differences; for linear Cα-ANM + ARAP versus linear Cα-ANM, 19 ties were excluded, leaving 14 positive and 2 negative nonzero differences.

**Table A7:** Dependence of retrospective minimum RMSD on the number of generated structures evaluated.

Structures Evaluated	Median RMSD Reduction (Å)	2.5–97.5% Paired-Subset Range (Å)	Resamples
1	0.173	−0.118 to 0.502	2000
5	0.13	0.00685 to 0.297	2000
10	0.135	0.0326 to 0.274	2000
20 (primary)	0.159	0.0598 to 0.31	2000
40	0.213	0.0962 to 0.366	2000
80	0.274	0.156 to 0.399	2000
160	0.315	0.237 to 0.398	2000
320	0.341	0.286 to 0.381	2000
640	0.345	0.328 to 0.366	2000
1600	0.346	—	—

Positive values indicate a lower retrospective minimum RMSD for BA-ARAP-NMA than for Linear Cα-ANM. The percentile range summarizes fixed paired structure subset resampling. At the full 1600-structure set, no subset resampling is possible, so the range and resample count are not applicable. The 20-structure analysis was the primary small-budget comparison. These two non-applicable entries are shown as em dashes (—).

**Table A8:** Cutoff sensitivity at requested amplitude 10 Å.

Endpoint	Cutoff (Å)	Requested Amplitude (Å)	Median Improvement	BCa 95% CI	Holm *p*
Adjacent-Cα distance MAE	12	10	0.625	0.553 to 0.657	3.49 × 10^−10^
RMSD to paired structure	12	10	0.203	0.0723 to 0.316	0.00376
Transition coverage	12	10	0.0215	0.0123 to 0.0281	0.00376
Virtual-angle MAE	12	10	4.74	4.04 to 5.01	3.49 × 10^−10^
Adjacent-Cα distance MAE	15	10	0.608	0.555 to 0.643	3.49 × 10^−10^
RMSD to paired structure	15	10	0.188	0.0652 to 0.339	0.0333
Transition coverage	15	10	0.0179	0.00757 to 0.0319	0.0333
Virtual-angle MAE	15	10	4.81	4.03 to 5.72	3.49 × 10^−10^
Adjacent-Cα distance MAE	18	10	0.612	0.562 to 0.678	3.49 × 10^−10^
RMSD to paired structure	18	10	0.156	0.00707 to 0.393	0.123
Transition coverage	18	10	0.0164	9.2 × 10^−4^ to 0.0366	0.123
Virtual-angle MAE	18	10	4.73	4.19 to 5.4	3.49 × 10^−10^

Positive values favor BA-ARAP-NMA. Estimates are based on the requested-amplitude cutoff grid at 10 Å and therefore differ from the matched-actual-displacement estimates in Table A3.

### Appendix A.4. Target-Free NOLB Comparison and Computational Cost

**Table A9:** Twelve-pair comparator set for the target-free NOLB analysis.

Protein System	Common Cα	Pair RMSD (Å)	Motion Class
Pyruvate phosphate dikinase (PPDK)	872	11.653	Large enzyme-domain motion
LeuT transporter	492	3.627	Alternating-access transport
GroEL chaperonin subunit	524	12.378	Chaperonin-domain rearrangement
Sarcoplasmic reticulum Ca2+-ATPase 1 (SERCA1a)	994	13.971	P-type ATPase transport cycle
PG9 antibody light chain	211	7.687	Immunoglobulin-domain reorientation
TAF1 tandem bromodomains	249	10.775	Tandem-domain reorientation
Dbh translesion DNA polymerase	341	5.873	Polymerase-domain rearrangement
Mechanosensitive channel MscS	261	6.918	Mechanosensitive-channel gating
CprK transcriptional regulator	225	15.843	Regulatory-domain rearrangement
Rous sarcoma virus integrase	216	14.416	Integrase-domain rearrangement
Neural cell adhesion molecule FNIII domains	196	8.610	Adhesion-domain reorientation
Human spindle-checkpoint protein Mad2	187	9.841	Topological fold switch

The 35 pairs were classified by common-Cα count (<200, 200–399, or ≥400) and pair RMSD (<7, 7–<12, or ≥12 Å). Each of the seven occupied groups contributed at least one pair. The remaining positions were allocated in proportion to group size, and MscS was included to represent mechanosensitive gating. Within each group, motion-class and family diversity were prioritized. Selection was completed before NOLB generation and did not use method performance. Full allocation details are provided in Supplementary File S1.

**Table A10:** Primary target-free BA-ARAP-NMA versus NOLB comparison using 20 structures per method.

Endpoint	Displacement (Å)	Median Improvement	BCa 95% CI	Pairs Favorable	Holm *p*
RMSD to paired structure	1	0.088	0.0315 to 0.145	10/12	0.0771
RMSD to paired structure	2	0.302	0.0807 to 0.358	10/12	0.0771
RMSD to paired structure	3	0.455	0.302 to 0.509	11/12	0.0381
RMSD to paired structure	5	0.837	0.476 to 1.12	11/12	0.0381
RMSD to paired structure	8	1.46	0.938 to 1.95	11/12	0.0381
RMSD to paired structure	10	1.39	0.936 to 2.29	11/12	0.0381
Transition coverage	1	0.00723	0.00323 to 0.0149	10/12	0.0771
Transition coverage	2	0.0237	0.0075 to 0.0469	10/12	0.0771
Transition coverage	3	0.0386	0.0276 to 0.0717	11/12	0.0381
Transition coverage	5	0.0739	0.0529 to 0.139	11/12	0.0381
Transition coverage	8	0.15	0.0949 to 0.218	11/12	0.0381
Transition coverage	10	0.179	0.0834 to 0.275	11/12	0.0381
Adjacent-Cα distance MAE	1	0.0747	0.0576 to 0.0918	12/12	0.00293
Adjacent-Cα distance MAE	2	0.188	0.135 to 0.225	12/12	0.00293
Adjacent-Cα distance MAE	3	0.301	0.224 to 0.339	12/12	0.00293
Adjacent-Cα distance MAE	5	0.568	0.47 to 0.74	12/12	0.00293
Adjacent-Cα distance MAE	8	1.03	0.864 to 1.16	12/12	0.00293
Adjacent-Cα distance MAE	10	1.29	1.04 to 1.4	12/12	0.00293
Virtual-angle MAE	1	1.42	1.02 to 1.98	12/12	0.00293
Virtual-angle MAE	2	2.82	2.42 to 4.32	12/12	0.00293
Virtual-angle MAE	3	4.21	3.76 to 6.35	12/12	0.00293
Virtual-angle MAE	5	7.83	6.18 to 10.4	12/12	0.00293
Virtual-angle MAE	8	11.3	7.97 to 13	12/12	0.00293
Virtual-angle MAE	10	14.4	9.14 to 17.5	12/12	0.00293

Positive values favor BA-ARAP-NMA. For RMSD and local-error metrics, the effect is NOLB minus BA-ARAP-NMA; for transition coverage, the effect is BA-ARAP-NMA minus NOLB. The local geometry values refer to the minimum-RMSD candidate in each fixed target-free set, not to the median geometry of all generated candidates. Supplementary File S1 contains Wilcoxon signed-rank analyses, Hodges–Lehmann shifts, and rank-biserial effects. It also reports candidate-budget sensitivity and results from the 2000 candidate-set resamples.

**Table A11:** End-to-end computational cost summary.

Procedure	Calculations	Median Time (s)	Min Time (s)	Max Time (s)	Median Memory (MB)	Min Memory (MB)	Max Memory (MB)
Linear Cα-ANM	70	14.6	5.10	66.2	—	—	—
BA-constrained modes	70	14.2	5.30	66.5	—	—	—
Linear Cα-ANM + ARAP	70	48.0	17.3	217	—	—	—
BA-ARAP-NMA	70	47.0	17.6	229	—	—	—
Complete four-way ablation calculation	70	124.8	45.7	593.6	94.7	75.3	512.2
Target-free NOLB protocol	24	151.8	103.1	828.3	22.1	17.4	96.0

Variant-specific wall times were recorded within each four-way ablation calculation. Peak resident memory was measured once for the complete calculation process and is therefore reported only for that calculation, not repeated as a variant-specific measurement. NOLB values include calibration, full-atom random-ensemble generation, and displacement filtering; its memory value is the peak process-tree resident set size. Accordingly, the variant-specific memory entries are shown as em dashes (—).

### Appendix A.5. Matched-Displacement and Branch-Level Robustness

Table A12 summarizes the sensitivity of the primary matched-displacement effects to stricter matching tolerances. Table A13 reports branch-level effect distributions and hierarchical resampling at the branch and protein-pair levels. Supplementary File S1 provides full-precision values, excluded-cell diagnostics, and generation scripts.

**Table A12:** Sensitivity of the primary complete BA-ARAP-NMA versus linear Cα-ANM matched-displacement results to the matching tolerance.

Displacement (Å)	Available Branches, 0.10–0.25 Å	Adjacent-Cα Distance-MAE Reduction (Å)	Virtual-Angle-MAE Reduction (°)	RMSD Reduction (Å)	Transition Coverage Increase
1	1321–1400	0.0538–0.0548 (35/35)	1.22–1.28 (35/35)	0.0158 (32–34/35)	0.00164–0.00169 (32–34/35)
2	1157–1400	0.116–0.122 (35/35)	1.99–2.08 (35/35)	0.0483–0.0702 (33/35)	0.00557–0.00662 (33/35)
3	1094–1400	0.178–0.191 (35/35)	2.79–2.80 (35/35)	0.123–0.131 (34–35/35)	0.0132–0.0146 (34–35/35)
5	1043–1399	0.334–0.343 (35/35)	3.84–4.19 (35/35)	0.181–0.199 (31–34/35)	0.0212–0.0226 (31–34/35)
8	1091–1399	0.525–0.541 (35/35)	4.66–4.92 (35/35)	0.364–0.367 (34/35)	0.0383–0.0420 (34/35)
10	1085–1400	0.665–0.667 (35/35)	4.88–4.95 (35/35)	0.521–0.527 (34–35/35)	0.0524–0.0553 (34–35/35)

Ranges summarize analyses using matching tolerances of 0.10, 0.15, 0.20, and 0.25 Å. Positive values favor BA-ARAP-NMA; values in parentheses give the range of favorable protein-pair counts. The branch column gives the minimum–maximum number of valid complete-method versus linear contrasts among the four tolerances. All 35 protein pairs and both input states remained represented at every tolerance. Primary 0.25 Å estimates and BCa intervals are reported in Table A3; complete values and matching diagnostics are provided in Supplementary File S1.

**Table A13:** Branch-level distributions and hierarchical resampling sensitivity at the branch and protein-pair levels for complete BA-ARAP-NMA versus linear Cα-ANM.

Endpoint	Displacement (Å)	Available Branches	Favorable Branches (%)	Pairs with Majority Favorable Branches	Hierarchical Median [2.5–97.5%]
Adjacent-Cα distance-MAE reduction (Å)	1	1400	99.1	35/35	0.0549 [0.0448 to 0.0653]
Adjacent-Cα distance-MAE reduction (Å)	2	1400	98.9	35/35	0.118 [0.0968 to 0.139]
Adjacent-Cα distance-MAE reduction (Å)	3	1400	98.9	35/35	0.188 [0.156 to 0.217]
Adjacent-Cα distance-MAE reduction (Å)	5	1399	99.2	35/35	0.336 [0.285 to 0.378]
Adjacent-Cα distance-MAE reduction (Å)	8	1399	98.9	35/35	0.546 [0.480 to 0.614]
Adjacent-Cα distance-MAE reduction (Å)	10	1400	98.0	35/35	0.666 [0.585 to 0.739]
Virtual-angle-MAE reduction (°)	1	1400	99.3	35/35	1.22 [0.975 to 1.46]
Virtual-angle-MAE reduction (°)	2	1400	99.8	35/35	2.09 [1.69 to 2.53]
Virtual-angle-MAE reduction (°)	3	1400	99.9	35/35	2.82 [2.23 to 3.41]
Virtual-angle-MAE reduction (°)	5	1399	99.6	35/35	3.93 [3.22 to 4.52]
Virtual-angle-MAE reduction (°)	8	1399	98.6	35/35	4.76 [4.09 to 5.50]
Virtual-angle-MAE reduction (°)	10	1400	97.6	35/35	5.02 [4.35 to 5.80]
RMSD reduction (Å)	1	1400	60.4	29/35	0.0176 [0.00542 to 0.0309]
RMSD reduction (Å)	2	1400	62.9	34/35	0.0590 [0.0274 to 0.0947]
RMSD reduction (Å)	3	1400	65.2	34/35	0.116 [0.0644 to 0.176]
RMSD reduction (Å)	5	1399	66.4	33/35	0.227 [0.129 to 0.346]
RMSD reduction (Å)	8	1399	68.2	34/35	0.391 [0.230 to 0.582]
RMSD reduction (Å)	10	1400	69.7	35/35	0.499 [0.283 to 0.711]
Transition Coverage increase	1	1400	60.4	29/35	0.00176 [0.000555 to 0.00328]
Transition Coverage increase	2	1400	62.9	34/35	0.00594 [0.00293 to 0.00989]
Transition Coverage increase	3	1400	65.2	34/35	0.0120 [0.00712 to 0.0187]
Transition Coverage increase	5	1399	66.4	33/35	0.0243 [0.0155 to 0.0356]
Transition Coverage increase	8	1399	68.2	34/35	0.0420 [0.0285 to 0.0560]
Transition Coverage increase	10	1400	69.7	35/35	0.0526 [0.0353 to 0.0688]

Effects are oriented so that positive values favor BA-ARAP-NMA. Branches are deterministic conditions defined by mode rank and orientation rather than independent biological replicates. The hierarchical sensitivity analysis resampled available branches within each input state and then resampled protein pairs across 10,000 replicates. All intervals were entirely favorable. Protein-pair-level BCa intervals in Table A3 remain the primary inferential results. Complete branch distributions and resampling records are provided in Supplementary File S1.
